# A Continuity Flow Based Tomographic Reconstruction Algorithm for 4D Multi-Beam High Temporal—Low Angular Sampling

**DOI:** 10.3390/jimaging7110246

**Published:** 2021-11-20

**Authors:** Axel Henningsson, Stephen A. Hall

**Affiliations:** Division of Solid Mechanics, Lund University, Ole Römers väg 1, 221 00 Lund, Sweden; stephen.hall@solid.lth.se

**Keywords:** tomography, 4D, temporal, dynamic, continuity equations

## Abstract

A mathematical framework and accompanying numerical algorithm exploiting the continuity equation for 4D reconstruction of spatiotemporal attenuation fields from multi-angle full-field transmission measurements is presented. The algorithm is geared towards rotation-free dynamic multi-beam X-ray tomography measurements, for which angular information is sparse but the temporal information is rich. 3D attenuation maps are recovered by propagating an initial discretized density volume in time according to the advection equations using the Finite Volumes method with a total variation diminishing monotonic upstream-centered scheme (TVDMUSCL). The benefits and limitations of the algorithm are explored using dynamic granular system phantoms modelled via discrete elements and projected by an analytical ray model independent from the numerical ray model used in the reconstruction scheme. Three phantom scenarios of increasing complexity are presented and it is found that projections from only a few (unknowns:equations > 10) angles can be sufficient for characterisation of the 3D attenuation field evolution in time. It is shown that the artificial velocity field produced by the algorithm sub-iteration, which is used to propagate the attenuation field, can to some extent approximate the true kinematics of the system. Furthermore, it is found that the selection of a temporal interpolation scheme for projection data can have a significant impact on error build up in the reconstructed attenuation field.

## 1. Introduction

X-ray tomography has become a standard experimental technique for revealing the internal 3D structure of objects. For static samples, 3D volume reconstruction from a set of 2D projections is a well researched area and algorithms such as FBP, ART, SART, SIRT, to name a few, have gained wide spread use, with optimised open source implementations easily available, c.f. [1,2]. Over the past decade, attention has been drawn towards dynamic tomography, with rapidly deforming or mutating sample volumes, c.f. [3]. The measured quantity from a tomography experiment usually comes in a pixelated format, as a photon count/intensity readout from a detector at discrete moments in time. The analytical form of these measurements with respect to time is not known and, commonly, the sought volume is reconstructed at individual instances of time. This imposes two competing requirements on the experiment, namely that (I) the studied object remains approximately constant over a measurement series and (II) that each measurement series be sufficiently sampled spatially to uniquely recover the object. For single beam geometries studying highly dynamic systems, characterised by continuous motion, these requirements may be difficult to fulfil simultaneously. Limitations include detector readout speed, low beam flux (resulting in a poor signal to noise ratio) and centrifugal and shear forces due to fast sample rotations. Although these issues can, to some extent, be resolved by hardware solutions [4], well regularised reconstruction algorithms have played an important role in recent advances, e.g., [5,6,7,8].

Attention towards multi-beam imaging systems has been increasing in recent years, e.g., [9,10,11,12,13,14,15]. These systems can acquire, simultaneously, a sparse set of 2D projections (∼10) of the studied sample, reducing the required sample rotation speed. For several specialised cases, such as steady flow in granular media [12] and weakly deforming solids [7], solutions in terms of hardware and algorithms have been found. Nevertheless, rotation is still required for samples undergoing large deformations through unsteady flow and the required rotation speed and associated centrifugal forces induced on the sample volume will finally limit what physical processes can and can not be studied. In cases when a sparse number of static projection angles is enough for the desired type of reconstruction, temporal resolution is instead only limited by the rate at which projections can be acquired. Compared to the synchrotron case of [3] this could push temporal resolution in 4D imaging by at least an order of magnitude (from hHz to kHz). Likewise, if used in conjunction with Free Electron Lasers, such as the European XFEL, temporal resolution in the MHz range could be within reach. In addition, reconstruction schemes targeting static projection data have an impact on a wider scene as they equip multi-source lab tomography devices with high speed imaging capability.

We investigate a tomographic reconstruction scheme that operates from a sparse angularly-static set of 2D projections, attempting to remove the need for rotations during studies of dynamic processes by providing a solution strategy that is less specialised compared to existing methods. Since such acquisitions are still performed only as pilot experiments, with limited experimental data available, we use numerical simulations to verify our method. By making use of the LIGGGHTS Discrete Elements Method (DEM) software [16], together with an independent analytical ray model, we establish a routine for generating physically realistic and challenging 4D phantoms of granular systems. Although the presented reconstruction scheme is not limited to granular systems, these phantoms serve to validate and illustrate our reconstruction method. As a side effect, the established phantom generation procedure could be used to evaluate the feasibility of granular flow experiments as well as to evaluate the accuracy of other reconstruction algorithms, specifically geared towards granular flow.

The proposed reconstruction method uses a single, initial, well-sampled reconstruction of the sample as the basis to track the time evolution of the sample. By enforcing the sample evolution to obey the continuity-flow differential equations with a prescribed reduced velocity basis, the problem is solved, despite the low angular sampling. Temporal propagation of the sought volume is carried out by numerical time integration using the Finite Volume Method resulting finally in a time series of 3D volumes of the density distribution of the studied object together with an associated series of 3D velocity fields. Similar to other tomographic reconstruction schemes our method relies on modelling X-ray attenuation by line integrals and is thus applicable to multi-beam setups which can establish such an approximation, either directly, or by some pre-processing of projections, to, for instance, make wavelength corrections, c.f. [14], or to address phase effects [17]. Additionally, as our method enforces mass conservation via the continuity-flow equation, we must require that no mass leaves the field of view of the imaging system, or, alternatively, that the boundary flow conditions of the system are well defined.

The paper is divided into two parts, where the first half of the paper consists of algorithmic details and the second part describes the numerical investigation for different phantom scenarios. The first part starts in Section 2 by describing the mathematical formulation of the inverse problem. In Section 3 and Section 4 we introduce the Finite Volume discretization and velocity basis used to solve the problem. The numerical time integration scheme used is laid out in Section 5 and, finally, the temporal interpolation scheme is described in Section 6. The first part of the paper is closed in Section 7 by an algorithm summary. The second part of the paper starts in Section 8 with a general description of how dynamic phantoms can be generated. Section 9 describes the phantoms used in the validation examples and the resulting reconstructions. In Section 10, we provide a discussion on our findings. Finally, the paper is closed with conclusions in Section 11.

## 2. Problem Formulation

Consider an X-ray attenuation experiment in which transmitted photon intensities are measured by a pixelated area detector. Let $I_{0k}$ denote the photon count of pixel number *k* when no attenuating sample is placed between source and detector. Similarly let $I_{1k}$ denote the photon count as the sample has been put in place. We define the absorbance along ray number *k* as

(1) $$A_{k}=\log\left(\frac{I_{0k}}{I_{1k}}\right).$$

Let f(x,t) be an attenuation function of space, $x={\left[\begin{array}{c} x,y,z \end{array}\right]}^{T}$, and time *t*. Assuming that Beer’s law holds we may establish an approximate line integral model of the measured absorbance at pixel *k* as

(2) $$A_{k}\left(t\right)=\int_{0}^{L_{k}}f\left(p_{k}+s{\hat{r}}_{k},t\right)ds,$$

where $p_{k}$ is the X-ray entry point at the sample boundary, *s* and $L_{k}$ scalars, ${\hat{r}}_{k}$ is the X-ray propagation direction and $p_{k}+L_{k}{\hat{r}}_{k}$ is the X-ray exit point at the sample boundary.

For acquisitions featuring sample rotation, both ${\hat{r}}_{k},\;p_{k}$ will be functions of time. In a multi-beam setup, when the sample is kept fixed in position, ${\hat{r}}_{k}$ and $p_{k}$ are static and the temporal evolution of $A_{k}\left(t\right)$ is driven solely by the internal dynamics of the studied sample. We seek to reconstruct *f* from *A* for the latter of these cases. However, as the number of measurements required to recover *f* uniquely is typically much larger than the available data, we must make some additional model assumptions. If the sample forms a closed system, the underlying mass density distribution of the sample, ρ(x,t), will follow the partial differential equations of continuity,

(3) $$\frac{\partial \rho }{\partial t}+\nabla^{T}\left(\rho u\right)=0,$$

where $u^{T}=\left[\begin{array}{ccc} u & v & w \end{array}\right]$ is a velocity field and $\nabla^{T}=\left[\begin{array}{ccc} \partial /\partial x & \partial /\partial y & \partial /\partial z \end{array}\right]$ is the divergence operator. The attenuation for X-rays is approximately proportional to the mass density of the sample, ρ≈cf, and thus, by dividing (3) with the unknown scalar *c*, we expect

(4) $$\frac{\partial f}{\partial t}+\nabla^{T}\left(fu\right)=0,$$

which provides us with an additional constraint partly bridging the gap of missing data.

Typically, when studying dynamic processes over short time spans, the experimental setup will involve a triggering system to initiate the desired internal sample processes. This also means that the initial state of the sample will be static prior to any multi-beam measurements taking place. Thus, we will further assume that it is possible to sample *f* fully for at least a single time point, $t_{0}$, by performing an initial classic single-beam, full-rotation tomography of the sample.

By time differentiation of (2) we have

(5) $$\frac{\partial A_{k}\left(t\right)}{\partial t}=\int_{0}^{L_{k}}\frac{\partial f(p_{k}+s{\hat{r}}_{k},t)}{\partial t}ds=\int_{R_{k}}\frac{\partial f}{\partial t}dr,$$

where we have introduced the right hand side shorthand integral notation where $R_{k}$ is the line segment defining ray number *k*. Inserting (4) into (5) and collecting our results we may now identify an initial value problem of the form

(6) $$\begin{array}{cc} \; & \frac{\partial A_{k}\left(t\right)}{\partial t}=-\int_{R_{k}}\nabla^{T}\left(fu\right)dr,\\ \; & \frac{\partial f}{\partial t}=-\nabla^{T}\left(fu\right),\\ \; & f\left(x,t_{0}\right)=f_{0}. \end{array}$$

We propose to reconstruct the sample attenuation, *f*, by finding solutions in time to (6).

## 3. Finite Volume Discretization

Analytical solutions to the continuity equations (3) for arbitrary density and velocity fields are not known. Numerically sophisticated Finite Volume Methods for propagating these equations in time do, however, exist. Below we derive a discretized format of the continuity equations needed for our algorithm. These results are standard and here included with the purpose of clarifying our notation and aid the reader not familiar with the Finite Volumes Method, for a general introduction to computational fluid dynamics c.f. [18].

**Figure 1:**
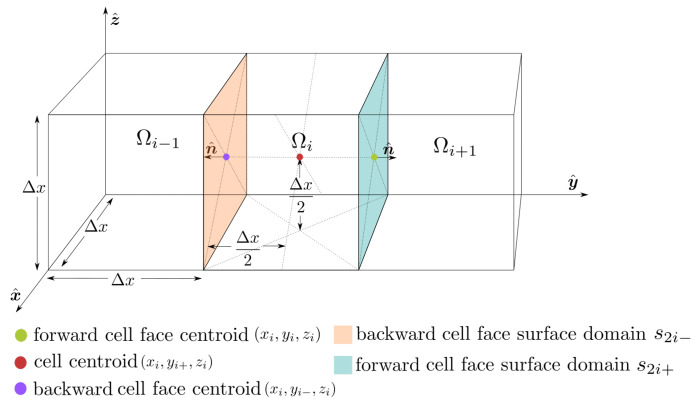
Schematic of the spatial Finite Volume discretization centered in *y* at cell number *i*. Each cubic cell is associated with six faces featuring normals aligned with the Cartesian ($\hat{x},\hat{y},\hat{z}$) coordinate system.

To make use of Finite Volume methods we must split space into identical cubic domains, $\Omega_{i}$, each with volume $\Delta x^{3}$ and cell faces aligned with a Cartesian coordinate system, as exemplified in Figure 1.

To further easily refer to quantities associated to cell centroids or cell face centroids we denote the cell centroid coordinate of cell number *i* as ($x_{i},y_{i},z_{i}$) and define

(7) $$\begin{array}{cc} \; & x_{i+}=x_{i}+\frac{\Delta x}{2},\;x_{i+1}=x_{i}+\Delta x,\\ \; & y_{i+}=y_{i}+\frac{\Delta x}{2},\;y_{i+1}=y_{i}+\Delta x,\\ \; & z_{i+}=z_{i}+\frac{\Delta x}{2},\;z_{i+1}=z_{i}+\Delta x. \end{array}$$

Furthermore, we denote the surface domain of any one cell as $S_{i}$, which is defined as the union of the six cell face surface domains;

(8) $$S_{i}=\sum_{d=1}^{d=3}\left(s_{di+}+s_{di-}\right),$$

where $s_{di+}$ and $s_{di-}$ denote the forward and backwards cell face surface domains in a dimension d=1,2,3 corresponding x=1,y=2,z=3.

Integrating (3) over a single cell yields

(9) $$\frac{\partial {\bar{\rho }}_{i}}{\partial t}=-\frac{1}{\Delta x^{3}}\int_{\Omega_{i}}\nabla^{T}\left(\rho u\right)dx,$$

where $\left(\bar{\cdot }\right)$ represents a cell average. Using the divergence theorem we may transform (9) into a surface integral of the form

(10) $$\frac{\partial {\bar{\rho }}_{i}}{\partial t}=-\frac{1}{\Delta x^{3}}\oint_{S_{i}}\rho u^{T}\hat{n}dS,$$

where $S_{i}$ is the surface of the element region $\Omega_{i}$ and $\hat{n}$ is the unit normal vector of this surface. We may now once again divide through with an unknown constant scaling factor *c* to represent (10) in terms of attenuation

(11) $$\frac{\partial {\bar{f}}_{i}}{\partial t}=-\frac{1}{\Delta x^{3}}\oint_{S_{i}}fu^{T}\hat{n}dS.$$

Using the fact that the cell is cubic and aligned with the coordinate axes we may rewrite (11) as

(12) $$\frac{\partial {\bar{f}}_{i}}{\partial t}=-\frac{1}{\Delta x^{3}}\sum_{d=1}^{d=3}\left(\int_{s_{di+}}fu_{d}ds-\int_{s_{di-}}fu_{d}ds\right).$$

The average density change in time is now easily identified as the sum of net fluxes measured at the cell interfaces. Many Finite Volume schemes for approximating these right hand side fluxes of (12) exist. Such schemes can produce excellent approximations to the per cell average temporal derivative $\partial \bar{f}/\partial t$. However, we seek to solve an initial value problem (6) involving not $\bar{f}$ but *f*. This distinction is not a problem for our purposes. In fact, due to the discrete nature of the measurements, $A_{k}$, any reconstruction of *f* will always have limited resolution. Typically, in tomographic reconstruction, *f* is assumed to be represented on a voxelated grid and numerical ray models, representing the integral operator $\int_{R_{k}}\left(\cdot \right)dr$, will already assume that *f* is piece-wise constant over a voxel. In state-of-the art projection algorithms, this assumption is a crucial component to maintain a computationally fast operator [2]. If we select the Finite Volume mesh to have cells that coincide with the voxels of our initial reconstructed function $f_{0}$ we may proceed by simply stating that the approximation $\partial \bar{f}/\partial t\approx \partial f/\partial t$ is valid whenever the ray model is to be executed upon ∂f/∂t. When executing the Finite Volume scheme and approximating $\partial \bar{f}/\partial t$, it will be important to distinguish *f* from $\bar{f}$ in the sense that the value *f* at some necessary evaluation coordinate in space must be extrapolated from $\bar{f}$.

To approximate the per cell temporal average attenuation evolution, $\partial \bar{f}/\partial t$, we have opted to use the central scheme by Kurganov and Tadmor [19]. This is a so called Monotonic Upstream-centered Scheme for Conservation Laws (MUSCL) which can provide accurate solutions in the presence of sharp discontinuities in the solution field *f*. This selected scheme can also be made to have a total variational diminishing (TVD) property if a suitable accompanying temporal integration scheme is selected. These two properties are especially desirable for dynamic systems where the constitutive particles exist in a solid state and undergo translations, rotations and deformations. In such a system, at the interface between constitutives, *f* may feature sharp gradients which must not be smoothed as a result of temporal integration. It is possible that simpler schemes, of lower order accuracy, may yield good results for many systems, but such a study is out of scope for this paper.

Explicitly, using the central scheme by Kurganov and Tadmor [19], the approximation scheme described above renders (12) in discrete form as

(13) $$\frac{\partial {\bar{f}}_{i}}{\partial t}\approx -\frac{1}{\Delta x}\sum_{d=1}^{d=3}\left(F_{di+}-F_{di-}\right).$$

Here the numerical fluxes at the cell faces are approximated as

(14) $$\begin{array}{cc} \; & F_{di-}=\frac{u_{di-}}{2}\left(f_{di-}^{R}+f_{di-}^{L}\right)-\frac{|u_{di-}|}{2}\left(f_{di-}^{R}-f_{di-}^{L}\right),\\ \; & F_{di+}=\frac{u_{di+}}{2}\left(f_{di+}^{R}+f_{di+}^{L}\right)-\frac{|u_{di+}|}{2}\left(f_{di+}^{R}-f_{di+}^{L}\right), \end{array}$$

where |·| denotes absolute value and $u_{di-}$ and $u_{di+}$ are the scalar velocity field component values, (u,v,w), at the *i*-th backwards and forwards cell face centroid coordinate in dimension *d*, i.e.,

(15) $$\begin{array}{cc} \; & u_{1i-}=u\left(x_{i-},y_{i},z_{i}\right),\;u_{1i+}=u\left(x_{i+},y_{i},z_{i}\right),\\ \; & u_{2i-}=v\left(x_{i},y_{i+},z_{i}\right),\;u_{2i+}=v\left(x_{i},y_{i+},z_{i}\right),\\ \; & u_{3i-}=w\left(x_{i},y_{i},z_{i+}\right),\;u_{3i+}=w\left(x_{i},y_{i},z_{i+}\right). \end{array}$$

The right ($f_{di+}^{R},f_{di-}^{R}$) and left ($f_{di+}^{L},f_{di-}^{L}$) extrapolated cell values are taken as

(16) $$\begin{array}{cc} \; & f_{di+}^{R}={\bar{f}}_{i+1}-\frac{\phi (r_{i+1})}{2}\left({\bar{f}}_{i+2}-{\bar{f}}_{i+1}\right),\\ \; & f_{di-}^{R}={\bar{f}}_{i}-\frac{\phi (r_{i})}{2}\left({\bar{f}}_{i+1}-{\bar{f}}_{i}\right),\\ \; & f_{di+}^{L}={\bar{f}}_{i}+\frac{\phi (r_{i})}{2}\left({\bar{f}}_{i+1}-{\bar{f}}_{i}\right),\\ \; & f_{di-}^{L}={\bar{f}}_{i-1}+\frac{\phi (r_{i-1})}{2}\left({\bar{f}}_{i}-{\bar{f}}_{i-1}\right), \end{array}$$

with

(17) $$r_{i}=\frac{{\bar{f}}_{i}-{\bar{f}}_{i-1}}{{\bar{f}}_{i+1}-{\bar{f}}_{i}}.$$

The super-bee limiter function ϕ [20] introduced in (16) is defined as

(18) $$\phi \left(r_{i}\right)=\left\{\begin{array}{cc} \; & 2\;\mathrm{if}\;{\bar{f}}_{i+1}-{\bar{f}}_{i}=0\\ \; & \max\left[0,\min\left(2r_{i},1\right),\min\left(r_{i},2\right)\right],\;\mathrm{otherwise}, \end{array}\right.$$

and serves to limit the gradient of *f* at strong discontinuities. Limiter functions are commonly used in higher order Finite Volume schemes to avoid spurious oscillations.

Before proceeding any further, to simplify notation, we introduce an operator FV[·] representing the action of the finite volume scheme on $\bar{f}$ and u together with a linear operator P[·] representing the action of a numerical ray model on $\bar{f}$,

(19) $$\mathrm{FV}\left[\bar{f},u\right]=-\frac{1}{\Delta x}\sum_{d=1}^{d=3}\left(F_{d+}-F_{d-}\right),$$

(20) $$P_{k}\left[\bar{f}\right]=\int_{0}^{L_{k}}f\left(p_{k}+s{\hat{r}}_{k},t\right)ds.$$

We use the ASTRA-toolbox [2] 3D-vector implementation of P[·]; this implementation features GPU acceleration via the Nvidia CUDA platform, which is necessary for computational tractability of the method.

With this notation we may write the modified initial value problem to solve as

(21a) $$\begin{array}{c} \frac{\partial A_{k}\left(t\right)}{\partial t}=P_{k}\left[\mathrm{FV}\left[\bar{f},u\right]\right], \end{array}$$

(21b) $$\begin{array}{c} \frac{\partial f}{\partial t}=\mathrm{FV}\left[\bar{f},u\right], \end{array}$$

(21c) $$\begin{array}{c} \bar{f}\left(x,t_{0}\right)={\bar{f}}_{0}, \end{array}$$

where the reader may note that we have now reformulated the problem in terms of cell average absorptivities, $\bar{f}$.

## 4. Velocity Representation

We must now define the velocity field to be evaluated at the cell face centroid coordinates over the Finite Volume mesh. Importantly, we need a parametrisation of u that can accurately represent the true velocity field of the system, and, at the same time, be fully recoverable through solving the intermediate tomographic Equation (21a) for a fixed time and fixed $\bar{f}$. We introduce a finite basis approximation of u as

(22) $$u\left(x,t\right)=\sum_{j=1}^{j=M}\alpha_{j}\left(t\right)\varphi_{j}\left(x\right),$$

with $\alpha_{j}^{T}=\left[\begin{array}{ccc} \alpha_{j} & \beta_{j} & \gamma_{j} \end{array}\right]$ featuring three independent velocity coefficient components. In this work, $\varphi_{j}\left(x\right)$, is defined on a tetrahedral mesh in a Finite Element type style. We consider element number *e*, connected to node number *j*, with an interior domain $E_{e}$, and define the linear interpolation as

(23) $$\varphi_{j}\left(x\right)=c_{0}^{\left(ej\right)}+c_{1}^{\left(ej\right)}x+c_{2}^{\left(ej\right)}y+c_{3}^{\left(ej\right)}z,\;x\in E_{e},$$

where the coefficients $c_{i}^{\left(ej\right)}$ are selected uniquely by requiring that $\varphi_{j}\left(x\right)=1$ at node *j* and $\varphi_{j}\left(x\right)=0$ for all other nodes in the mesh.

We stress again that the basis should be selected to fulfil two competing requirements, namely, that of being recoverable from (21a) for fixed *t* and $\bar{f}$, while, at the same time, being generic enough to represent the true velocity field well throughout the duration of the experiment. If no prior information on the velocity representation is known, this fundamentally limits the scope of the method to study phenomena where a generic basis selection, such as the above Finite Element basis, can capture the kinematics of the sample. Depending on the number of available data it could be difficult to reconstruct processes with high total variation in the velocity field. On the contrary, we note that the method puts no restriction on the attenuation function, $\bar{f}$, itself which could have extremely rich spatial patterns with sharp gradients and a high total variation.

The reader may now be tempted to think that we have only transferred the problem of reconstructing the scalar field $\bar{f}$ from too few data to the problem of reconstructing the vector field u from the same data and equations. If this was the case, we would be at a loss, since we have but multiplied the number of unknowns by a factor of three, leaving us worse than we started out. However, we must recall that Equation (21a) is fundamentally constrained by (I) $\bar{f}$ being known and (II) the operator FV[·] representing continuous flow. In other words the action of u upon *f* is severely restricted. For instance, a velocity acting on an evacuated cell in the Finite Volume mesh will have no impact on the updated attenuation field *f*. Similarly, it is not possible for u to increase the attenuation value of a single cell in the mesh without also affecting the attenuation of the neighbours of that cell. We raise this point to stress that the presented initial value problem is fundamentally different from solving the tomography equations themselves.

## 5. Time Integration and CFL Number

To propagate $\bar{f}$ in time through (21) we need to select a temporal integration scheme. To achieve TVD properties we have selected a Third-order Strong Stability Preserving Runge–Kutta (SSPRK3) scheme [21].

Let D be an operator yielding the right hand side of the partial differential equation in (21b),

(24) $$D\left[t_{l},\bar{f}\right]=\frac{\partial \bar{f}}{\partial t}.$$

The SSPRK3 scheme is, thus, described by

(25) $$\begin{array}{cc} \; & k_{1}=D\left[t_{l},\bar{f}\left(t_{l}\right)\right],\\ \; & k_{2}=D\left[t_{l}+\Delta t,\bar{f}\left(t_{l}\right)+\Delta tk_{1}\right],\\ \; & k3=D\left[t_{l}+\frac{\Delta t}{2},\bar{f}\left(t_{l}\right)+\Delta t\left(\frac{k_{1}}{4}+\frac{k_{2}}{4}\right)\right],\\ \; & \bar{f}\left(t_{l+1}\right)=\bar{f}\left(t_{l}\right)+\frac{\Delta t}{6}\left(k_{1}+k_{2}+4k_{3}\right). \end{array}$$

Importantly the selection of Δt is ultimately limited by the Courant–Friedrichs–Lewy (CFL) condition, which states that the CFL number, *C*, must fulfil the inequality

(26) $$C=\frac{(|u_{x}|+|u_{y}|+|u_{z}|)\Delta t}{\Delta x}\leq 1.$$

Especially this means that when the cells of the Finite Volume discretization are selected to match the lengths of the detector pixels the attenuation field, *f*, must feature sub-pixel motion for meaningful results to be obtained.

When executing $D[t_{l},\bar{f}]$, the intermediate step of solving (21a) for u must yield a solution that does not violate (26) such that, when u is used in (21b) by the flow model $\mathrm{FV}[\bar{f},u]$, stability is preserved. Note that (21a) is an upper bound on the flow field, in practice, to maintain TVD for the scheme, *C* will have to be smaller than unity.

We have implemented $D[t_{l},\bar{f}]$ by first solving (21a) in a least squares sense for the velocity basis coefficients $\alpha_{j}$ after which $\mathrm{FV}[\bar{f},u]$ can be executed using the resultant velocity field. Specifically, we solve

(27) $${\mathrm{Argmin}}_{\alpha_{j}}\sum {\left(P_{k}\left[\mathrm{FV}\left[\bar{f},u\right]\right]-\frac{\partial A_{k}\left(t\right)}{\partial t}\right)}^{2}$$

using the L-BFGS-B minimisation scheme [22]. To do this, we must compute the partial derivatives of the flow model with respect to the free variables; analytical expressions for these necessary gradients, $\partial \mathrm{FV}\left[\bar{f},u\right]/\partial \alpha_{j}$, are presented in Appendix B. The resulting solution, $\alpha_{j}^{*}$, is then brought back into the feasible domain by setting

(28) $$\alpha_{j}=\left\{\begin{array}{c} \frac{\alpha_{j}^{*}}{C(\alpha_{j}^{*})},\;\mathrm{if}\;C\left(\alpha_{j}^{*}\right)>1\\ \alpha_{j}^{*},\;\mathrm{otherwise}, \end{array}\right.$$

where

(29) $$C\left(\alpha_{j}^{*}\right)=\frac{(|\alpha_{jx}|+|\alpha_{jy}|+|\alpha_{jz}|)\Delta t}{\Delta x}.$$

We can here also mention that, when solving (27) repeatedly for a sequence of time points, it can be useful to start the minimisation at the previously located minima to speed up convergence. If the velocity varies slowly between time steps optimal solutions may, thus, be found in a few iterations.

## 6. Temporal Measurement Interpolation Scheme

To execute the Runge–Kutta time integration scheme, the projected rates, $\partial A_{k}\left(t\right)/\partial t$, must be available. We must now decide how to interpolate $A_{k}\left(t\right)$ from the available discrete data series ($A_{k}\left(t_{0}\right),(A_{k}\left(t_{1}\right),...,A_{k}\left(t_{T}\right)$). We have selected a quadratic interpolation scheme represented in a local basis such that

(30) $$A_{k}\left(t\right)=a_{0}^{\left(l\right)}q^{2}+a_{1}^{\left(l\right)}q+a_{2}^{\left(l\right)},\;t\in \left[\begin{array}{c} t_{l},t_{l+1} \end{array}\right],$$

where $t_{l}$ is any time at which $A_{k}$ was measured. The polynomial coefficients, $a_{i}^{\left(l\right)}$, are determined by the data of the interval, $A_{k}\left(t_{l}\right),A_{k}\left(t_{l+1}\right)$, and a continuity requirement on $\partial A_{k}/\partial t$ such that

(31) $$\begin{array}{cc} \; & a_{0}^{\left(l\right)}t_{l}^{2}+a_{1}^{\left(l\right)}t_{l}+a_{1}^{\left(l\right)}=A_{k}\left(t_{l}\right),\\ \; & a_{0}^{\left(l\right)}t_{l+1}^{2}+a_{1}^{\left(l\right)}t_{l+1}+a_{2}^{\left(l\right)}=A_{k}\left(t_{l+1}\right),\\ \; & \lim_{t\to t_{l}+}\frac{\partial A_{k}}{\partial t}=\lim_{t\to t_{l}-}\frac{\partial A_{k}}{\partial t}. \end{array}$$

To close out the system of equations we require initially that

(32) $$\frac{\partial A_{k}}{\partial t}{|}_{t_{0}}=0,$$

i.e., we require that the sample starts from a static state. The interpolation scheme of (30) allows for evaluation of both $A_{k}\left(t\right)$ as well as ∂A(t)/∂t at arbitrary time-points, *t*, and is the lowest order polynomial interpolation scheme that gives continuous ∂A(t)/∂t. Since the projection operator P[·] is linear, (30) implies that we expect $\bar{f}$ to evolve quadratically over a single timestep. If this approximation holds, the third order RK scheme could theoretically produce an exact integration using a timestep $\Delta t=t_{l+1}-t_{l}$.

In practice, considering model errors from both ray and flow models, as well as noise in the data, the integration will not be exact. Thus, the updated state $\bar{f}\left(t_{l+1}\right)$ will not match the acquired projections $P\left[\bar{f}\left(t_{l+1}\right)\right]\neq A\left(t_{l+1}\right)$ perfectly. Nonetheless, when integration is performed, the left hand side of (21a) is requested as a function of the current state $\bar{f}\left(t_{l}\right)$. We must therefore make an interpretation of ∂A(t)/∂t for attenuation functions that do not represent the true states of the sample, and thus do not lie on the interpolated path defined by the measurements. Naively, we could set ∂A(t)/∂t to the original derivative found in the quadratic interpolation scheme. This will, however, be sensitive to errors in $\bar{f}$, as any offset from the original data path will be preserved and amplified throughout time integration. To combat this we may perform, before each integration step, a re-interpolation, introducing the current projected state of $\bar{f}$ into the data series, replacing the current measurement. This ensures an interpolation path that connects the current erroneous state with the true data points. As a result the projection derivatives, ∂A(t)/∂t, on the interval $t_{l}$ to $t_{l+1}$ will be modified such that the projection of the updated state $\bar{f}\left(t_{l+1}\right)$ has a tendency to return to the data path. Note that the re-interpolation is introduced to modify ∂A(t)/∂t and that the data points, A(t), described by the re-interpolation are meaningless in themselves.

**Figure 2:**
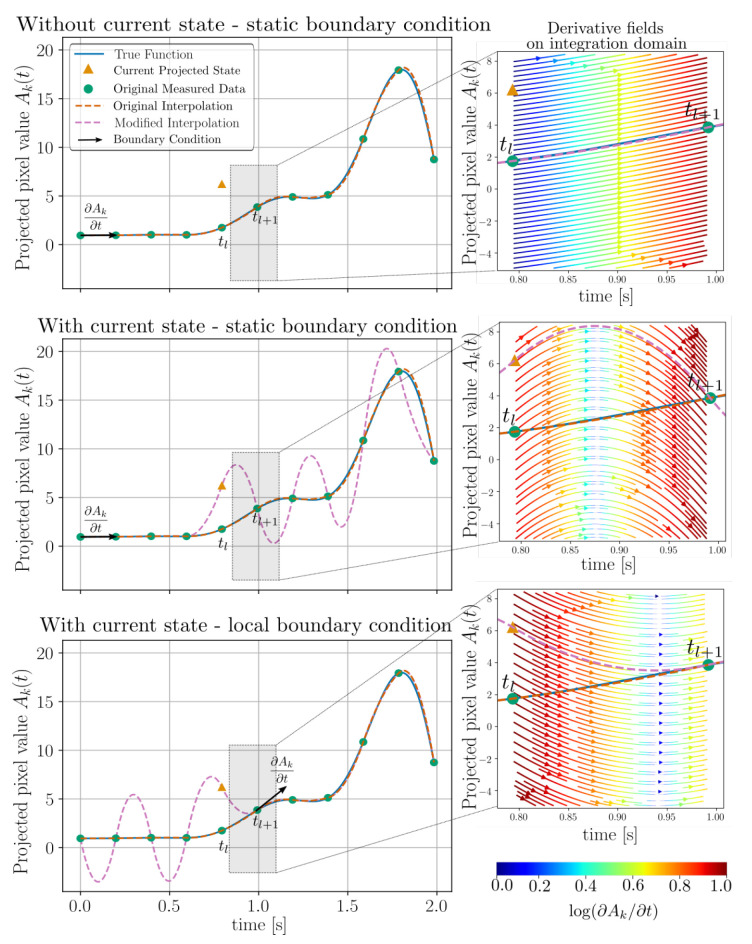
Resulting quadratic re-interpolation using; current projected state and a local boundary condition (**bottom**), current projected state using a static boundary condition (**middle**), disregarding current projected state and using static boundary condition, i.e., using the original interpolation (**top**). As integration is executed from $t_{l}$ to $t_{l+1}$ the derivatives used in the RK3 scheme will be sampled from the fields illustrated in the right column.

To illustrate these concepts we have considered three different re-interpolation strategies as illustrated in Figure 2. Two of these strategies makes use of the current projected state of $\bar{f}$ while the third naively neglects this current state. Considering a situation when $\bar{f}$ is reconstructed at a current time point $t=t_{l}$ and we seek to integrate forward in time to $t=t_{l+1}$ the three strategies may be summarised as follows:

1. Interpolation using only the original data, i.e., neglecting the current projected state of $\bar{f}\left(t_{l}\right)$ during interpolation. The boundary condition for interpolation is applied statically (regardless of $t_{l}$) at $t_{0}$ as in Equation (32). This interpolation scheme will naively disregard any information related to the current state $t_{l}$.
2. Re-interpolation using original data with the exception of replacing the current data point at $t_{l}$ with the current projected state of $\bar{f}\left(t_{l}\right)$. The boundary condition for interpolation is again applied statically (regardless of $t_{l}$) at $t_{0}$ as in Equation (32).
3. Re-interpolation using original data with the exception of replacing the current data point at $t_{l}$ with the current projected state of $\bar{f}\left(t_{l}\right)$. The boundary condition for interpolation is now applied locally to $t_{t+1}$ as
  (33) $$\frac{\partial A_{k}}{\partial t}{|}_{t_{l+1}}=\frac{\partial A_{k}^{*}}{\partial t}{|}_{t_{l+1}}$$
  where $A_{k}^{*}$ represents an interpolation using strategy (1.). This means that starting from $t=t_{l+1}$ the re-interpolation is forced to join the original ($t=t_{0}$) interpolation.

To visualise the meaning of the three above interpolation strategies, without yet evaluating which is more accurate, we provide the illustration of Figure 2.

**Figure 3:**
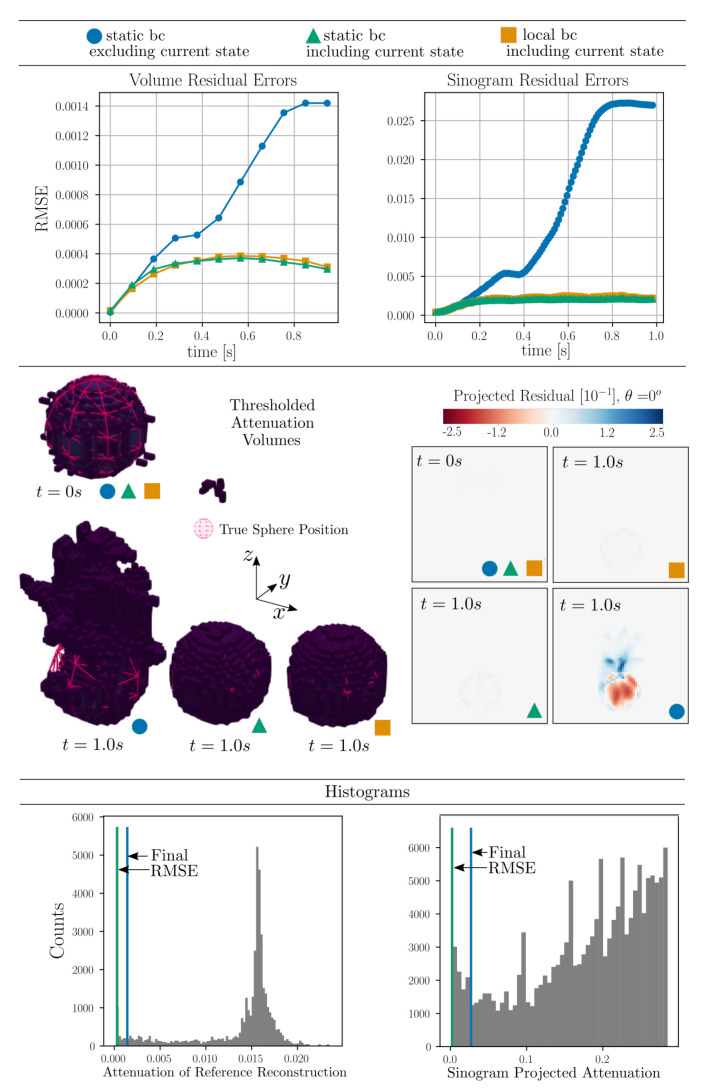
Reconstructions by Algorithm 1 using three different re-interpolation schemes. The static bc (green) and local bc (orange), which correspond to the second and third row of Figure 2 are seen to produce better reconstructions than the original static bc scheme (blue). The sinogram residuals (parallel beam at angle θ=0) and thresholded volumes (at 1% of their maximum value) are illustrated for the start and endpoints in time. The true sphere position is rendered on top of the reconstructed attenuation volumes as a cerise mesh. Histograms over per-cell volume attenuation and sinogram projected attenuation values are shown in the bottom row (clipped at 1% of their maximum value). The final RMSE for each of the three interpolation schemes are illustrated as vertical lines in these histograms.

To illustrate the impact of integration scheme upon the reconstructed time series we consider a single spherical particle moving along a straight line in 3D space. We assign the particle the velocity function

(34) $$v\left(t\right)={\left[\begin{array}{ccc} 0 & 0 & -z_{0}\sin\left(\pi t/t_{end}\right) \end{array}\right]}^{T},$$

where $z_{0}$ is the initial position of the sphere and *t* goes from 0 to $t_{end}$. The CFL number now becomes variable in time, as

(35) $$C\left(t\right)=z_{0}sin\left(\pi t/t_{end}\right)dt/dx,$$

reaching a prescribed maximum $z_{0}dt/dx=0.5$ at $t_{end}/2$. Using simulated projections from 5 separate angles (described in detail in Section 8 and Section 9) we may define a simulated data time series. Reconstruction from these projection data is illustrated in Figure 3 where the root mean squared error of both volume ($\bar{f}$) as well as projections ($P[\bar{f}]$) are shown as functions of time. By acquiring full simulated reference scans at multiple selected time points and performing, from these, classical tomographic reconstructions, the volume residuals can be analysed. To provide some comparative value to the RMSE, we present, in Figure 3, also histograms over the volume and sinogram values clipped at 1% of their maximum values.

Figure 3 indicates that it is desirable to include the current projected model state into the interpolation, as this creates resilience against error build up in the reconstruction, as the time integration will then be forced to return to the data path. In the following we select to use the local boundary condition scheme (bottom row Figure 2 and orange markings Figure 3). Although the selected re-interpolation scheme features some desirable properties, we do not claim to have an optimal selection. A deeper exploration of re-interpolation schemes is, however, out of scope for this paper.

## 7. Algorithm Summary

In the previous sections, we have described the numerical computations involved in the proposed time series reconstruction scheme. To make it clear in what sequence these operations are performed, we give a complete algorithm summary (Algorithms 1 and 2) in the form of pseudo-code below. Our current implementation of the algorithm is openly available at https://github.com/AxelHenningsson/contomo, accessed on 14 April 2021. This package also includes the modules used for phantom simulation, which are described in Section 8.
**Algorithm 1** Time series reconstruction scheme.**Input**: $f_{0}$, P[·], $t_{0},t_{1},t_{2},..$, $A\left(t_{0}\right),A\left(t_{1}\right),A\left(t_{2}\right),..$$t_{l}\leftarrow t_{0}$$\bar{f}\left(t_{l}\right)\leftarrow f_{0}$$\alpha_{j}^{init}\leftarrow 0$**while**$\;t_{l}<t_{end}\;$**do** Replace measurement at $t_{l}$ by current state, $A\left(t_{l}\right)\leftarrow P\left[\bar{f}\left(t_{l}\right)\right]$ Interpolate measurements sequence $A\left(t_{0}\right),A\left(t_{1}\right),A\left(t_{2}\right),..$ by Equation (30) Define $ALG_{2}\left[\cdot ,\cdot \right]$ by Algorithm 2 with current interpolation, A(t), and $\alpha_{j}^{init}$ $k_{1},\alpha_{j}^{1}\leftarrow ALG_{2}\left[t_{l},\bar{f}\left(t_{l}\right)\right]$ $k_{2},\alpha_{j}^{2}\leftarrow ALG_{2}\left[t_{l}+\Delta t,\bar{f}\left(t_{l}\right)+\Delta tk_{1}\right]$ $k_{3},\alpha_{j}^{3}\leftarrow ALG_{2}\left[t_{l}+\frac{\Delta t}{2},\bar{f}\left(t_{l}\right)+\Delta t\left(\frac{k_{1}}{4}+\frac{k_{2}}{4}\right)\right]$ $\bar{f}\left(t_{l}\right)\leftarrow \bar{f}\left(t_{l}\right)+\frac{\Delta t}{6}\left(k_{1}+k_{2}+4k_{3}\right)$ $t_{l}\leftarrow t_{l}+\Delta t$ $\alpha_{j}^{init}\leftarrow \alpha_{j}^{3}$**end while****Output**: attenuation, $\bar{f}$, and velocity, ***u***, at series of time points, $t_{0},t_{1},t_{2},..$

**Algorithm 2** Velocity recovery step (subroutine of Algorithm 1).

**Input**: *t*, $\bar{f}$

$u^{init}\leftarrow \sum_{j=1}^{j=M}\alpha_{j}^{init}\varphi_{j}\left(x\right)$

$\alpha_{j}^{*}\leftarrow {\mathrm{Argmin}}_{\alpha_{j}}\sum {\left(P_{k}\left[\mathrm{FV}\left[\bar{f},u\right]\right]-\frac{\partial A_{k}\left(t\right)}{\partial t}\right)}^{2}$ by L-BFGS-B and inital guess $u^{init}$
**for** j=1,2,..,M **do**
 $C_{j}\leftarrow \left(\right|\alpha_{jx}^{*}\left|+\right|\alpha_{jy}^{*}\left|+\right|\alpha_{jz}^{*}\left|\right)\Delta t/\Delta x$ **if** $C_{j}>=1$ **then**  $\alpha_{j}\leftarrow \alpha_{j}^{*}/C_{j}$ **else**  $\alpha_{j}\leftarrow \alpha_{j}^{*}$ **end if**
**end for**


$u\leftarrow \sum_{j=1}^{j=M}\alpha_{j}\varphi_{j}\left(x\right)$



$\frac{\partial \bar{f}}{\partial t}\leftarrow \mathrm{FV}\left[\bar{f},u\right]$

**Output**: $\frac{\partial \bar{f}}{\partial t}$, $\alpha_{j}$



## 8. Framework for Phantom Data Generation

To provide a challenging and realistic phantom test case for the proposed reconstruction algorithm, featuring physically realistic motion, we have simulated ensembles of spherical particles by the Discrete Element method, as implemented in LIGGGHTS [16]. The LIGGGHTS software package enables time series characterisation of a wide range of kinematic data on the individual particles included in a simulation run. For this work, DEM simulations featuring mixtures of uniform density spherical particles with varying radii have been performed. To create projections from the ensemble state data available from the DEM, we have computed the integral (2) analytically utilising the fact that the constitutive particles are spherical. Detailed derivations of the resulting integral expression can be found in Appendix A; here we only present the final used projection equations.

**Figure 4:**
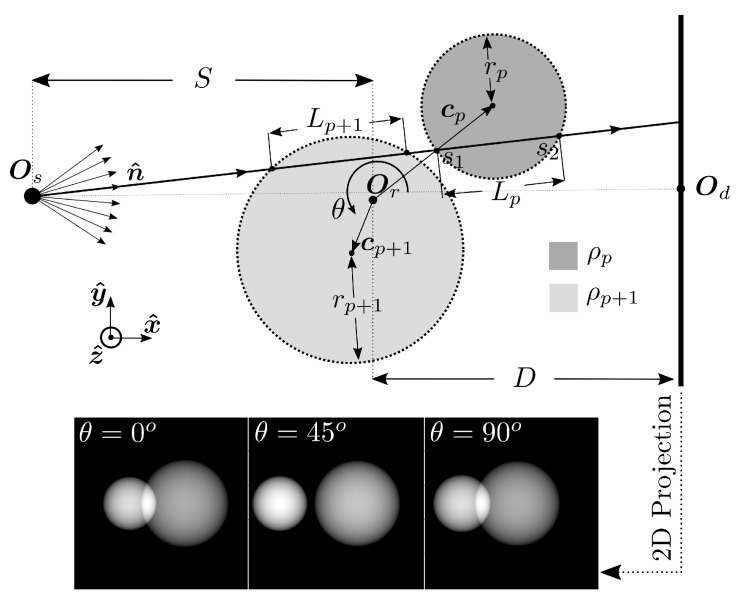
Top down 2D schematic of ray sphere intersection considering a point like X-ray source at $O_{s}$ and two illuminated spheres with centroids at $c_{p}$ and $c_{p+1}$.

Let us consider a set of particles centred around the origin, $O_{r}$, in a Cartesian coordinate system. Let further a particle be indexed by p=1,2,..,P and illuminated by a point like X-ray source at $O_{s}$ as schematically illustrated in Figure 4.

The projected attenuation for a given point in the image plane, ($x_{d},y_{d}$), and sample rotation, θ, is then associated to an X-ray propagating along the unit vector $\hat{n}$ as

(36) A(xd,yd)=∑p=1p=P2ρpReSp,

where

(37) $$\begin{array}{cc} \; & S_{p}={\left({\hat{n}}^{T}\left(O_{s}-c_{p}\right)\right)}^{2}-O_{s}^{T}O_{s}+\\ \; & c_{p}^{T}\left(2O_{s}-c_{p}\right)+r_{p}^{2}, \end{array}$$

and

(38) $$O_{s}=R_{z}\left[\begin{array}{c} -S\\ 0\\ 0 \end{array}\right],\;O_{d}=R_{z}\left[\begin{array}{c} D\\ 0\\ 0 \end{array}\right],$$

(39) $$\hat{n}=\frac{1}{\sqrt{{\left(D+S\right)}^{2}+x_{d}^{2}+y_{d}^{2}}}R_{z}\left[\begin{array}{c} D+S\\ x_{d}\\ y_{d} \end{array}\right].$$

where *S* and *D* are the source to rotation axis and detector to rotation axis distances, respectively, $x_{d},y_{d}$ are the *x* and *y* coordinates of the detector ray intersection point and $R_{z}$ is a 3 × 3 rotation matrix rotating a vector negatively around the *z*-axis. In this setting the line connecting the X-ray source, $O_{s}$, with the detector origin, $O_{d}$, is parallel to $\hat{x}$, i.e., the optical axis of propagation is along $\hat{x}$. The angle θ describes a positive sample rotation around $\hat{z}$. Further, $\rho_{p}$ is the sphere attenuation, $r_{p}$ the sphere radii, $c_{p}$ the sphere centroid coordinate and Re[·] denotes real value. Here we have assumed that the detector is perfectly mounted, i.e., ${\left(O_{d}-O_{s}\right)}^{T}\hat{z}=0$.

A single detector pixel value, $A_{k}$, of the detector can be assigned by (36) using the pixel centroid coordinate. However, to better mimic real measurements conditions, we may consider that the measured detector values are in fact integral measures over the surface domains of the pixels. In this paper, we account for this effect by, for each pixel, evaluating (36) at 9 separate locations located on an equidistant grid within the pixel square and assigning the value of pixel number *k* as the average of these 9 samples.

## 9. Numerical Simulations

Three separate phantom scenarios have been investigated. The first of these represents a three-particle system featuring prescribed large, nonlinear helical translations paths. In contrast to the predefined motion paths, the second two phantoms were generated by DEM simulations including hundreds of particles with motion initiated and driven by an impactor making contact upon a particle bed. Simulation and reconstruction parameters for the three phantom scenarios are presented in Table 1. The resulting reconstructions generated by Algorithm 1 are presented in Section 9.1 and Section 9.2 together with comparisons against ground truth reference reconstructions. For completeness, additional comparisons to reconstructions obtainable by existing iterative reconstruction methods are presented in the Supplementary Material. These additional comparisons were performed using the open source library tomopy [1] and features comparison to a compressed sensing technique similar to [23,24,25].

Using an NVIDIA Quadro P2000 for ray tracing and a single Intel(R) Core(TM) i7-8700K CPU@3.70GHz for all other calculations reconstruction was executed in 11.5 ± 0.2 s per time-step for all presented phantom scenarios. It is noteworthy that the algorithm scales linearly in both the number of time-steps as well as the number of voxels in the discretization and angular projections used. If a locally supported basis is used the number of basis functions have little to no impact on the compute time but primarily affect the memory usage of the algorithm.

### 9.1. Three Body Helical Motion System

We define a helical motion path by introducing the sphere centroid coordinate functions,

(40) $$\begin{array}{cc} \; & x=v_{xi}\sin\left(2\pi t\right)-t_{xi},\\ \; & y=v_{yi}\cos\left(2\pi t\right)-t_{yi},\\ \; & z=v_{zi}t-t_{yi}, \end{array}$$

where $v_{xi}$ and $v_{yi}$ bound the $\hat{x}$-$\hat{y}$ plane velocities, $v_{zi}$ is the particle velocity along the helix rotation axis ($\hat{z}$) and $t_{xi},t_{yi},t_{zi}$ define the initial position of the sphere centroid. For this example, we define a three particle system as in Table 2, which corresponds to a fixed central sphere surrounded by two spheres revolving in opposite directions.

The initial attenuation function, ${\bar{f}}_{0}$, provided for Algorithm 1 was produced by simulating an initial full projection set with Nπ projections equally spaced in the angular interval $\theta \in \left[\begin{array}{cc} 0^{{}^{\circ}} & 180^{{}^{\circ}} \end{array}\right]$, where *N* was the number of pixels along the detector side length. From the reference projection set an initial volume was reconstructed by solving the least squares residual equations,

(41) $${\mathrm{Argmin}}_{\bar{f}}\sum_{k}{\left(P_{k}\left[\bar{f}\right]-A_{k}\right)}^{2},$$

using L-BFGS-B [22] with a maximum number of allowed iterations equal to *N* and a maximum number of line searches equal to 25. The Finite Volume mesh was selected to align with the reconstructed voxel grid, resulting in a cubic mesh domain and cubic cells.

**Figure 5:**
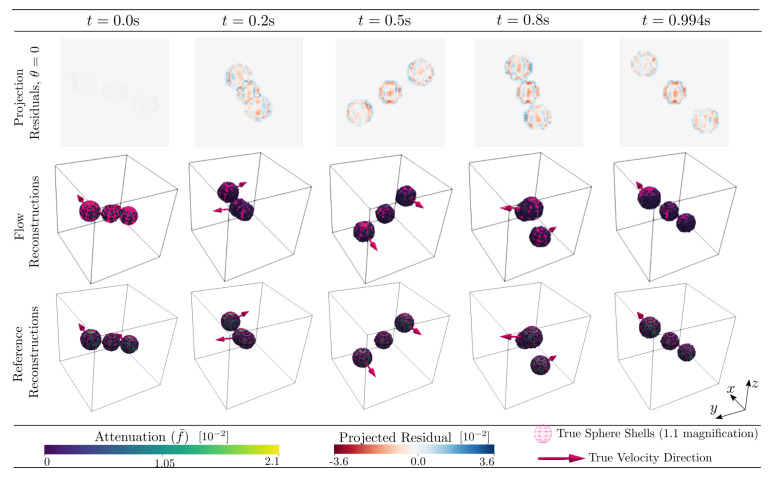
Reconstructed per-cell average 3D attenuation, $\bar{f}$, and projected residuals for the 3-particle system phantom undergoing helical motion. The bottom row illustrates the reference reconstructions from a full set of projections. The middle row illustrates the reconstruction from 5 projections using Algorithm 1. The top row shows the projection residual at $\theta =0^{{}^{\circ}}$ which corresponds to projecting along the *x*-axis into the *y*-*z*-plane. The 3D volume reconstructions are rendered by a fixed cut-off threshold (5% of maximum cell value) and shown for varying times as indicated by the legends. The reconstructions are overlayed with their corresponding input particle sphere shells with a 1.1 magnification for visibility. The true input velocities for the moving spheres are illustrated as 3D arrows in the renderings. Input parameters for simulation and reconstruction are described in Section 9.

Using Algorithm 1, reconstruction from the five projection angles described in Table 1 was performed. The resulting time-series of cell values was thresholded at 5% of the maximum cell value found throughout the time-series. In the middle row of Figure 5 these results are illustrated at five selected time-points. For comparison, the analytical particle positions (Equation (40)) and propagation directions have been overlayed on the cell attenuation values. The top row of Figure 5 shows the corresponding projected residuals between the analytical projections provided as input to Algorithm 1 and the numerical projections of the discretised reconstruction produced by Algorithm 1. This residual is an important measure of how well the reconstruction respects the input data. Finally, the bottom row of Figure 5 depicts reference reconstructions, generated independently through the same procedure described above for the initial attenuation function, ${\bar{f}}_{0}$. These reference reconstructions represent a best-case scenario, i.e., when full angular information of the sample is possible to be perfectly attained at each time-step without motion blurring.

**Figure 6:**
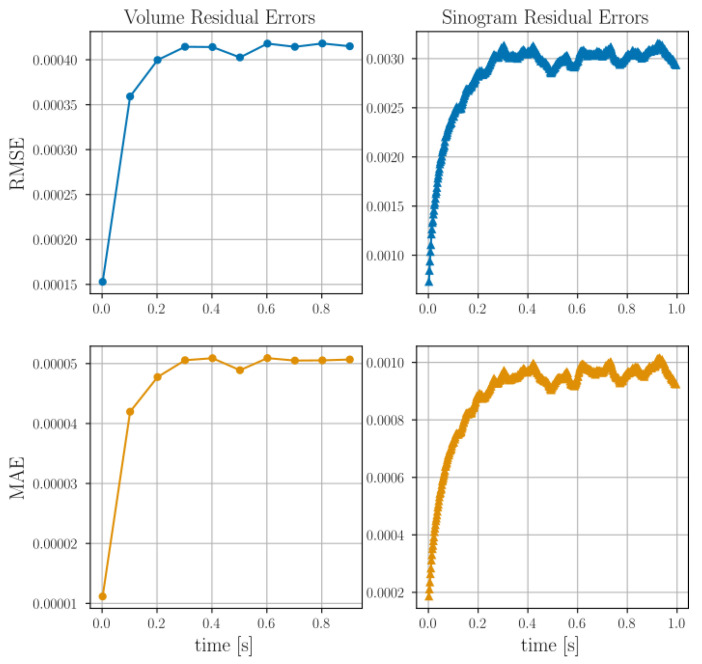
Root mean squared error (top row) and mean absolute error (middle row) for the attenuation residual field and the projected sinograms of the helical motion phantom shown in Figure 5. The residual fields at each time point are defined by subtracting the reconstructed attenuation value from the target value.

To quantify the error in the reconstructions, the real space and sinogram residuals were analysed over the full time-series. We define the root mean squared errors (RMSE),

(42) $$\mathrm{RMSE}=\sqrt{\sum_{i=1}^{i=n}\frac{r_{i}^{2}}{n}},$$

and the mean absolute errors (MAE),

(43) $$\mathrm{MAE}=\sum_{i=1}^{i=n}\frac{|r_{i}|}{n},$$

where a total of *n* residuals, $r_{i}$, are included in the sums. In this setting $r_{i}$ can be either the per cell average value of $\bar{f}$ compared between the reconstruction by Algorithm 1 and the reference tomography reconstructions, or, alternatively, $r_{i}$ may be the residuals between the numerically projected values of $\bar{f}$ and the ground truth simulated projections. The evolution in time of the RMSE and MAE for the helical motion phantom is presented in Figure 6.

**Figure 7:**
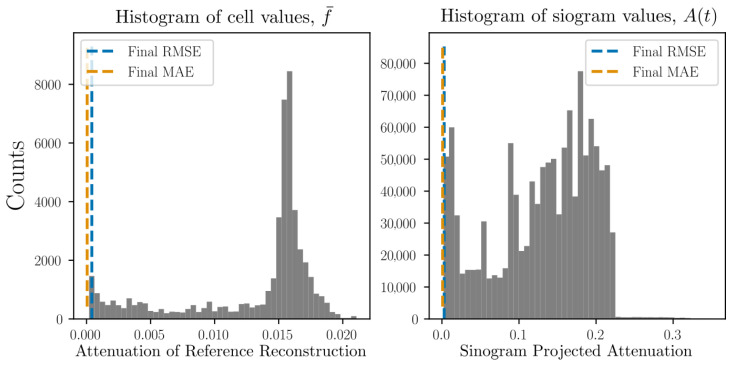
Histograms of the per pixel/per voxel distributions found in the reference volumes and ground truth sinograms of helical motion phantom shown in Figure 5. The RMSE and MAE found at the final reconstructed time point is marked in the corresponding histograms as dashed lines.

To put the magnitude of the RMSE and MAE in relation to the imaged system, histograms over the reference reconstructions and ground truth sinograms are presented in Figure 7 together with RMSE and MAE found at the final timestep.

**Figure 8:**
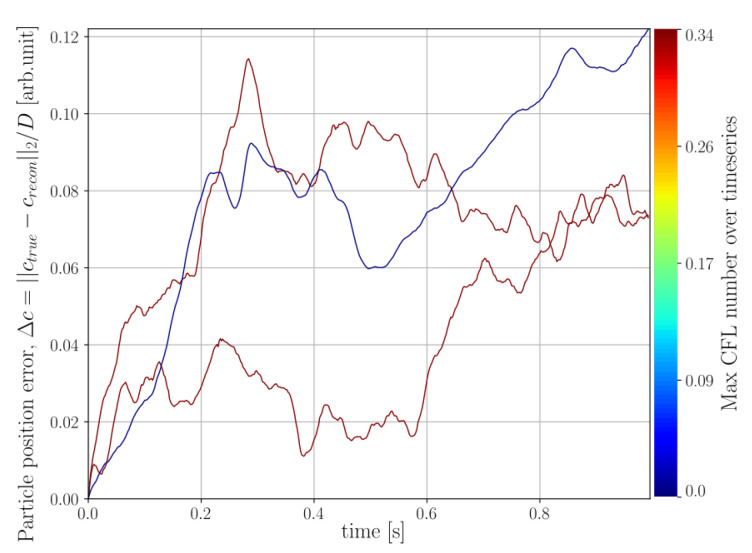
Distance error between true ($c_{true}$) and predicted ($c_{recon}$) sphere centroids normalised by sphere diameter (*D*) for the helical three body system depicted in Figure 5. Each line in the plot represents the error of a single sphere and has been colored by its corresponding maximum CFL number recorded at any instance in the time series.

To investigate how the velocity field reconstructed by the sub-iteration of Algorithm 1 relates to the true kinematics of the system, particle tracking has been performed using the initial true sphere positions and the reconstructed velocity field was used to predict the sphere trajectories. The normalised error between the true and reconstructed sphere centroids is defined as

(44) $$\Delta c=\left|\right|c_{recon}-c_{true}{\left|\right|}_{2}/D,$$

where $c_{recon}$ and $c_{true}$ denotes reconstructed and true sphere centroids, respectively, and *D* is the sphere diameter. The coordinate $c_{recon}$ was produced by RK4 integration and interpolating for intermediate velocities assuming quadratic motion trajectory over a single time step. The velocity at a given time step for a given sphere was defined by averaging over the sphere volume, using the current centroid, $c_{recon}$, together with the sphere radii. By the definition of (44), an error less than unity corresponds to overlap between the true and reconstructed sphere volumes. The errors Δc for the helical motion phantom are presented in Figure 8, where the error of each individual particle has been coloured its corresponding maximum CFL number found at any time during the simulation.

### 9.2. DEM Simulations

**Figure 9:**
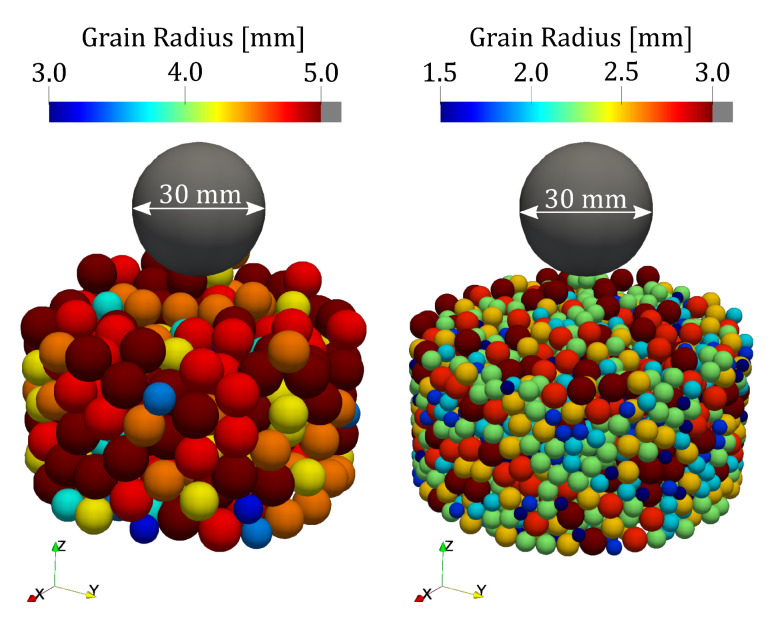
DEM simulations featuring gravity impact on particle beds for large (left) and small (right) grain size distributions. The gravity-driven impactor is the grey sphere above the grain ensemble.

Two DEM simulations were run featuring a total of 300 and 2200 particles, respectively. The radius-size distribution and initial geometry of the two simulations are depicted in Figure 9. The simulations featured the impact of a dense, large, spherical, gravity-driven impactor on a grain ensemble confined by a cylindrical tube. The LIGGGHTS input files, specifying the input parameters of the simulation, are provided in the Supplementary Materials.

**Figure 10:**
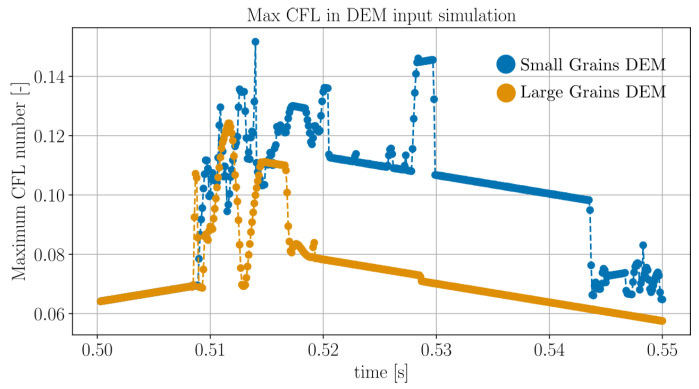
Time evolution of the maximum Courant–Friedrichs–Lewy (CFL) number for the two DEM simulation corresponding to Figure 9.

The maximum CFL number found for any sphere in the DEM simulation at each integration timepoint is depicted in Figure 10. It can be seen that the small-grains DEM features, in general, grains with higher velocities than the large-grains DEM and that it is feasible to reconstruct the motion of either system, as the CFL number is always less than unity for any grain at any time.

### 9.3. DEM Simulation—Large Grains

**Figure 11:**
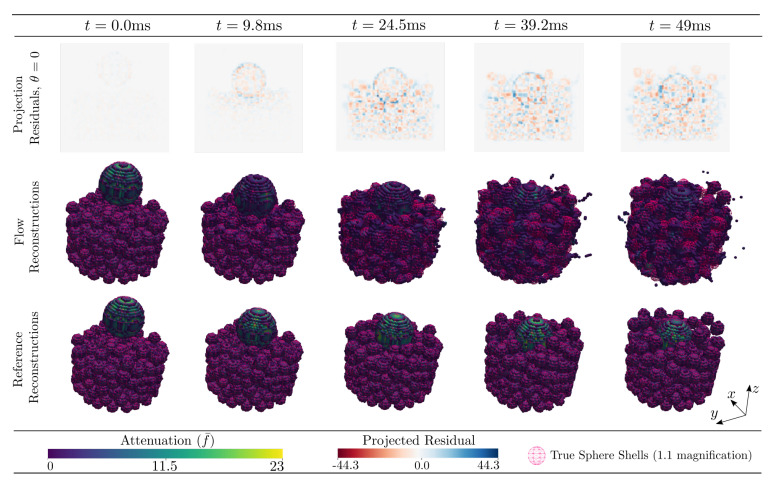
Reconstructed per cell average 3D attenuation, $\bar{f}$, and projected residuals for DEM phantom featuring impact upon a granular bed. The bottom row illustrates the reference reconstructions from a full set of projections. The middle row illustrates the reconstruction from 5 projections using Algorithm 1. The top row shows the projection residual at $\theta =0^{{}^{\circ}}$ which corresponds to projecting along the *x*-axis into the *y*-*z*-plane. The 3D volume reconstructions are rendered by a fixed cut-off threshold (5% of maximum cell value) and shown for varying time as indicated by the legends. The reconstructions are overlayed with their corresponding input particle sphere shells with a 1.1 magnification for visibility. Input parameters are described in Section 9.

The attenuation reconstructions by Algorithm 1 of the DEM phantom featuring large grains are found in Figure 11, which is arranged in the same way as Figure 5 with the thresholded results of Algorithm 1 presented in the central row.

**Figure 12:**
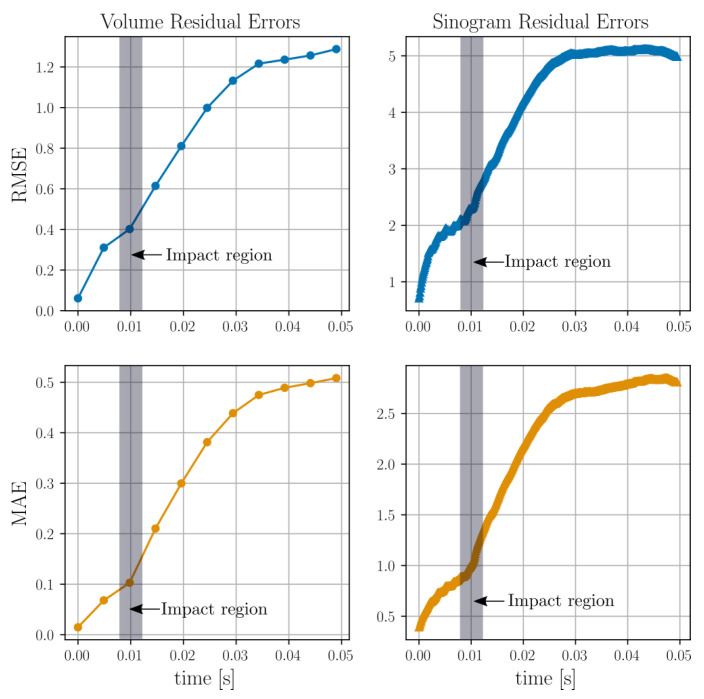
Root mean squared error (top row) and mean absolute error (bottom row) for the attenuation residual field and the projected sinograms of the DEM particle simulations shown in Figure 11.

The time evolutions of the RMSE and MAE, as defined in Equations (42) and (43), are presented in Figure 12. The moment the impact makes contact with the granular bed, instantiating motion therein, is marked in grey in the four subplots.

**Figure 13:**
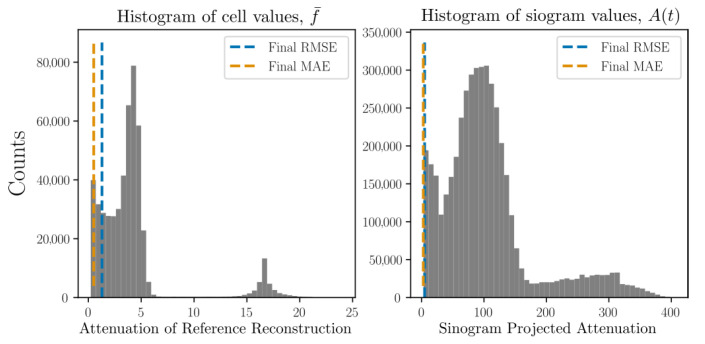
Histograms of the per pixel/per voxel distributions found in the reference volumes and ground truth sinograms. The RMSE and MAE found at the final reconstructed time point is marked in the corresponding histograms as dashed lines.

To put the RMSE and MAE in relation to the imaged system, histograms over the phantom attenuation and projection values, analogue to those shown in Figure 7, are presented in Figure 13.

**Figure 14:**
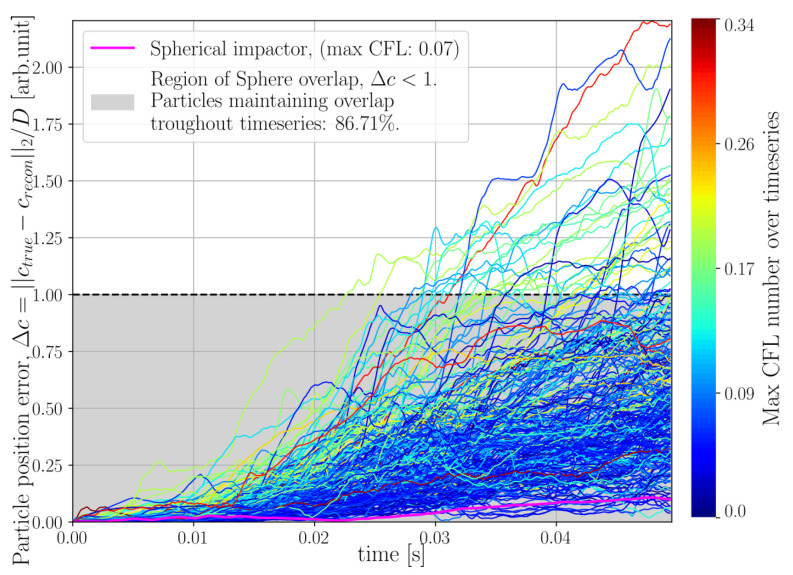
Distance error between true ($c_{true}$) and predicted ($c_{recon}$) sphere centroids normalised by sphere diameter (*D*) for the DEM simulation depicted in Figure 11. Each line in the plot represents the error of a single sphere and has been colored by its corresponding maximum CFL number recorded at any instance in the time series. The spherical impactor has been marked by an off-range color (magenta) for visibility.

Finally, for the DEM simulation featuring large grains, the result of velocity tracking is presented in Figure 14. The particle position error of the impactor has been marked in an off-coloured cerise, as opposed to the errors of the particle ensemble, which have been coloured by their corresponding maximum CFL found throughout the time-series. At the final timestep, it was found that 86.71% of the predicted particle positions still maintained overlap with their corresponding true positions. We remind the reader that a particle position error >1 means that the predicted and true position of a sphere has a zero overlap. From the colouring of the error curves, it is seen that particles with higher maximum CFL numbers tend to also produce higher particle position errors.

### 9.4. DEM Simulation—Small Grains

**Figure 15:**
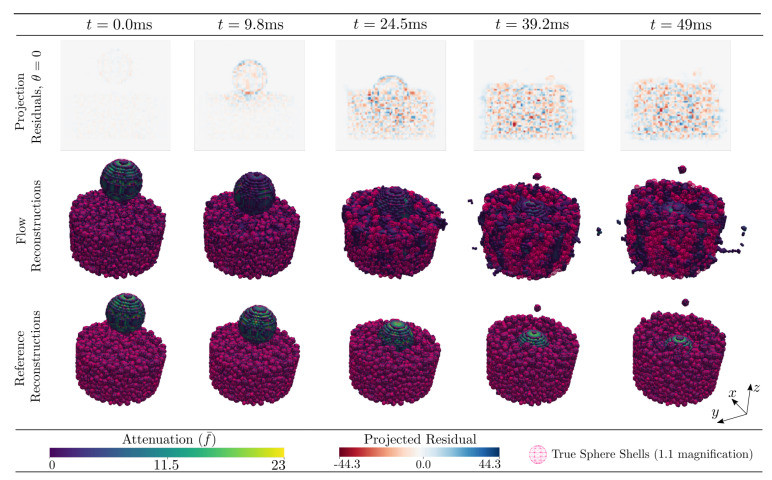
Reconstructed per cell average 3D attenuation, $\bar{f}$, and projected residuals for a challenging DEM phantom scenario featuring impact upon a granular bed. The bottom row illustrates the reference reconstructions from a full set of projections. The middle row illustrates the reconstruction from 5 projections using Algorithm 1. The top row shows the projection residual at $\theta =0^{{}^{\circ}}$ which corresponds to projecting along the *x*-axis into the *y*-*z*-plane. The 3D volume reconstructions are rendered by a fixed cut-of threshold (5% of maximum cell value) and shown for varying time as indicated by the legends. The reconstructions are overlayed with their corresponding input particle sphere shells with a 1.1 magnification for visibility. Input parameters are described in Section 9.

For the DEM featuring small grains the attenuation reconstructions by Algorithm 1 are found in Figure 15.

**Figure 16:**
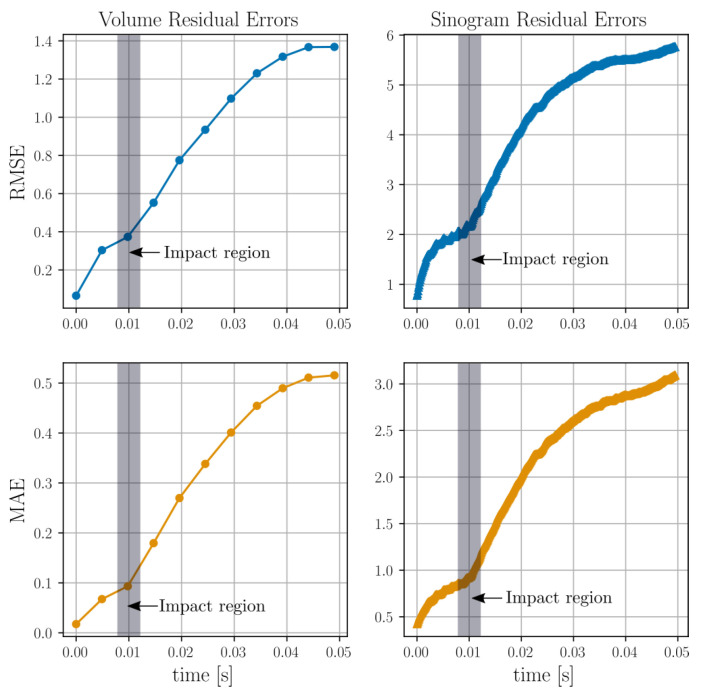
Root mean squared error (top row) and mean absolute error (middle row) for the attenuation residual field and the projected sinograms of the DEM particle simulations shown in Figure 15. The residual fields at each time point are defined by subtracting the reconstructed attenuation value from the target value.

Again, the time evolutions of the RMSE and MAE are presented in Figure 16.

**Figure 17:**
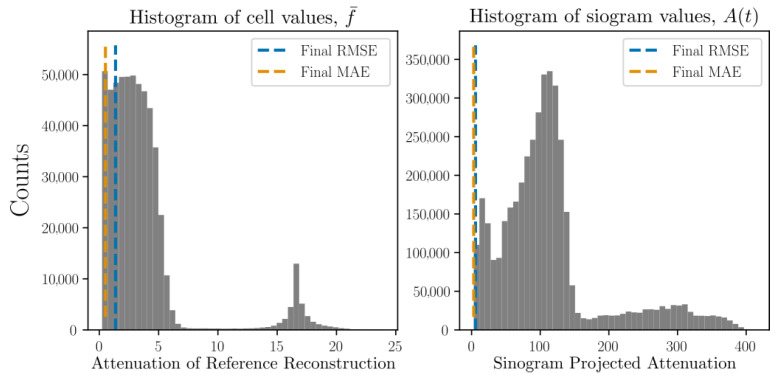
Histograms of the per pixel/per voxel distributions found in the reference volumes and ground truth sinograms of DEM particle simulations shown in Figure 15. The RMSE and MAE found at the final reconstructed time point is marked in the corresponding histograms as dashed lines.

Histograms over the reference reconstructions and ground truth sinograms are presented Figure 17 together with RMSE and MAE found at the final timestep.

**Figure 18:**
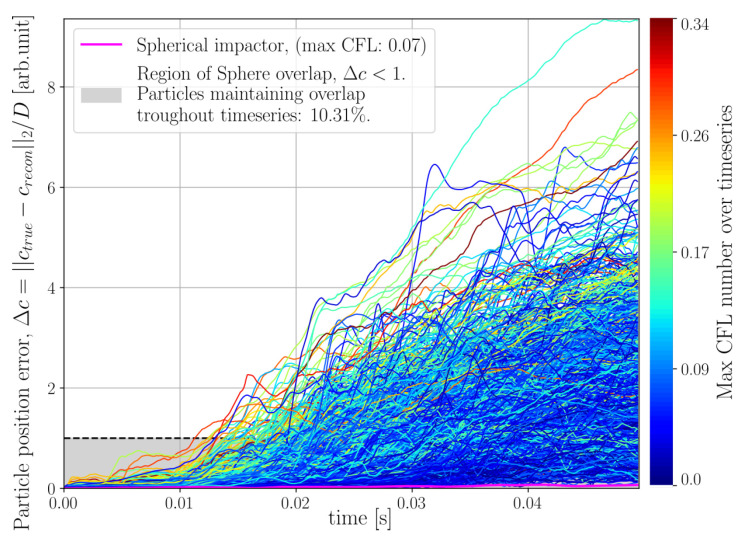
Error in Euclidean distance between true ($c_{true}$) and predicted ($c_{recon}$) sphere centroids normalised by sphere diameter (*D*) for the challenging DEM simulation depicted in Figure 15. Each line in the plot represents the error of a single sphere and has been colored by its corresponding maximum CFL number recorded at any instance in the time series. The spherical impactor has been marked by an off-range color (magenta) for visibility.

Velocity tracking was performed in the same way as previously described and the resulting particle error evolution is presented in Figure 18. In this case it was found that only 10.31% of the predicted particle positions still maintained overlap with their corresponding true positions at the final timestep.

## 10. Discussion

The projection residuals in the top row of Figure 5 display an initial, close-to-zero error followed by a rapid evolution and (possibly) stabilisation, with typical values of about two times that of the sphere attenuation. As the constitutive spheres have a diameter of several voxels/cells (>10) this represents a relative projected error much less than unity. The error magnitudes can be assessed further by Figure 7, where it is seen in the left histogram that the final MAE and RMSE in the real space reconstructions are far lower than the interior sphere values (peak in the histogram). This is also reflected in the 3D renderings of Figure 5, where the reconstructed thresholded volume fits well to the overlaid true particle positions and is visually very close to the reference reconstructions. The initial close-to-zero projected residual (as opposed to a zero residual) present at $t_{0}$ is due to analytical and numerical ray model discrepancy. In a best-case scenario, Algorithm 1 would propagate $\bar{f}$ in time without introducing any additional errors other than those that are inherent in the ray model. In Figure 6, an error build up to a few times this inherent error is, however, observed. Interestingly, this error build up seems to stabilise after about 100 timesteps. One might be tempted to think that this error cannot be attributed to a poor spatial velocity basis representation, as the same phenomenon can be observed in Figure 3 where the motion is uniform in $\hat{z}$ and, thus, perfectly represented by the finite basis expansion. One should however remember that, although the true underlying attenuation function may feature a velocity field that is uniform, the velocities needed to optimally update the discretised version of *f* may very well not be uniform. It is, therefore, likely that the discrepancy between true and discretised attenuation field, together with a finite basis expansion of the velocities, is driving the observed error. This would also explain why the error plateaus after some initial iterations, as then the best possible dynamic sphere representation allowed by the velocity expansion and continuity requirement has been reached. This inherent error would then indeed be expected to be higher than that of the best possible static sphere representation allowed by a classical tomographic reconstruction, where each individual cell value may be changed arbitrarily and independently of neighbouring cells.

Examination of Figure 5, Figure 6 and Figure 7, in comparison to Figure 11, Figure 12 and Figure 13 and Figure 15, Figure 16 and Figure 17, indicates better attenuation reconstructions by Algorithm 1 for the three-body system than for the multi-grain cases. These three examples serve to illustrate the impact of the velocity basis coarseness in relation to the true motion of the system. The three-body system features spheres that are both large and well separated in space compared to the dimension of the velocity basis element size, and it is possible for the basis expansion to take on fields that are close to those of the true underlying system. When the particle density grows successively in the DEM simulations the velocity basis discretisation must be made finer to capture rapid spatial velocity variations. Since the number of basis functions is kept fixed, however, it becomes increasingly more challenging for the algorithm to find velocity fields that yield good updates to the attenuation field. This explains how the largest RMSE and MAE errors are found in the small grains DEM phantom (Figure 15 and Figure 16) while the larger grain distribution DEM phantom (Figure 11 and Figure 12) have a reduced error and the three-body system (Figure 5 and Figure 6) has a close to zero RMSE and MAE. However, even in the most challenging test case, which features more particles than velocity basis functions (see Table 1), the attenuation field reconstruction depicted in Figure 15 still displays a reasonable distribution of the bulk mass of the system and is even able to capture the ejection of a single constitutive grain from the ensemble. We stress here that, apart from the initial conditions ($f\left(t_{0}\right)=f_{0}$), the algorithm has no access to sample specific prior information, which is a result of the attempt to formulate a generic solution strategy. Additional prior information on the kinematics of the sample could even further improve the reconstruction quality and reduce the number of needed velocity basis functions and projections. The most obvious of such constraints lies in the selection of the velocity basis itself limiting what type of motion that is permitted.

We note that, in the DEM impact scenarios (Figure 12 and Figure 16), the MAE and RMSE are seen to increase more rapidly from the moment the impactor makes contact with the granular bed (as marked in the figures). Before the moment of impact, the errors associated with the reconstruction of the static bed would be expected to only be attributed to the inherent previously discussed errors, which is reflected in the MAE and RMSE indicating plateauing prior to impact.

Examining the velocity fields produced by the sub-iteration of Algorithm 1, we see in Figure 8 that the motion of the three particle system is preserved throughout the full time-series with an error in the size of some small fraction of a sphere radius. We stress that the velocities produced by Algorithm 1 are optimised, not to capture the kinematics of the system, but to propagate the current reconstructed grid of attenuation cell values, through a Finite Volume model, to match the measured rate of the sinogram. From an algorithmic perspective, the velocities are but a vehicle to find allowable updates to the attenuation volume, which is the sought output from the algorithm. This means, in particular, that historical errors introduced into the attenuation volume, due to, for instance, finite discretisations or model approximations, may be corrected for later by introducing spurious velocities. Nevertheless, the results observed for the three-body system shows that these artificial velocities can capture the true kinematics of the system. Turning to the same metric for the DEM simulations we see that the velocities produced for the DEM with larger grains (Figure 14) better preserve the sphere positions in time compared to the small grains distribution (Figure 18). It is interesting to note that some correlation between high CFL numbers for the spheres and high position errors seem to be present. In particular, for the small grains DEM, the kinematics of the system are poorly captured, which may be partly explained by high CFL numbers and partly by the discretisation of attenuation and velocity field being coarse in comparison to the constitutive of the system. However, the exact conditions under which Algorithm 1 reliably reproduces the kinematics of the studied system remains a question for further study.

## 11. Conclusions

An algorithm for reconstruction of spatiotemporal attenuation fields, geared towards rotation-free, fast, multi-beam X-ray imaging, has been developed and numerically tested using an independent analytical ray model for phantom generation. The algorithm was found to produce reconstructions from a sparse set of projections (unknowns: equations > 10) with errors in the order of magnitude of those expected from classical full angle range tomography. Furthermore, it was found that the velocity fields produced by the algorithm sub-iterations can, under the right conditions, approximate the kinematics of the imaged system well. The proposed algorithm opens the door to unveiling the 3D evolution of physical processes unfolding at timescales currently inaccessible with existing techniques. In summary, the algorithm constrains the possible solutions by assuming conservation of the total linear X-ray attenuation within the field of view. For systems where no phase transformations are present, this is identical to enforcing mass conservation.

The presented algorithm opens for a rich survey and optimisation of parameter selections, including, but not limited to, velocity basis selection, Finite Volume scheme, time integration scheme and temporal interpolation scheme of the measurements. Improvements to the computational aspects of the algorithm, providing fast parallel implementations would also be desirable. To further evaluate the robustness of the algorithm for real data applications, the impact of X-ray phase propagation and detector noise in the measurement series are deemed to be key. Beyond these considerations, application to real, multi-beam, data could reveal further avenues of improvement. The proposed algorithm aims towards a generic as possible reconstruction scheme for multi-beam data. With this in mind, we suggest that the algorithm may serve as a basis to solve more advanced problems via the introduction of sample specific constraints.

## Figures and Tables

**Table 1:** Simulation and reconstruction parameters used in three phantom scenarios constructed to test Algorithm 1. For the given DEM phantoms the impactor radii (15 mm) is excluded from the given sphere radii range as well as the sphere count.

	Helical Motion	DEM Large Grains	DEM Small Grains
No. spheres	3	300	2200
Sphere radii range	[100.0–111.11] mm	[3.25–5.0] mm	[1.5–3.0] mm
Simulation duration	t∈[0,1000] ms	t∈[0,49.8] ms	t∈[0,49.8] ms
Projection readout speed	2 ms	0.1 ms	0.1 ms
No. sampled time points	501	499	499
Beam geometry	in plane parallel	in plane parallel	in plane parallel
Projection angles	−75${}^{\circ }$, −35${}^{\circ }$, 0${}^{\circ }$, 35${}^{\circ }$, 75${}^{\circ }$	−75${}^{\circ }$, −35${}^{\circ }$, 0${}^{\circ }$, 35${}^{\circ }$, 75${}^{\circ }$	−75${}^{\circ }$, −35${}^{\circ }$, 0${}^{\circ }$, 35${}^{\circ }$, 75${}^{\circ }$
Detector dimensions (pixels)	64 × 64	64 × 64	64 × 64
Detector pixel size	15.625 mm	1.5625 mm	1.5625 mm
No. velocity basis elements	661	1983	1983
Element enclosing sphere radii	[72.91, 147.42] mm	[4.91–9.75] mm	[4.91–9.75] mm
No. FVM cells	64 × 64 × 64	64 × 64 × 64	64 × 64 × 64
FVM cell size (Δx)	15.625 mm	1.5625 mm	1.5625 mm
RK integration time step	2 ms	0.1 ms	0.1 ms
Max L-BFGSG-B iterations	20	20	20
Max L-BFGSG-B line searches	25	25	25

**Table 2:** Parameters of three particle helical motion system, interpreted through Equation (40).

Particle Number *i*	1	2	3
$v_{xi}$ [m/s]	2/7	2/7	0
$v_{yi}$ [m/s]	2/7	−2/7	0
$v_{zi}$ [m/s]	3/20	−3/20	0
$t_{xi}$ [m]	0	0	0
$t_{yi}$ [m]	0	0	0
$t_{zi}$ [m]	9/64	−9/64	0

## Data Availability

The data presented in this study can be openly reproduced using the open source code: https://github.com/AxelHenningsson/contomo, accessed on 14 April 2021. The data is also available upon reasonable request to the corresponding author.

## Supplementary Materials

The following are available at https://www.mdpi.com/article/10.3390/jimaging7110246/s1, Text S1 and Figures S1–S3: Reconstruction comparison to existing iterative tomography methods, Text S2: LIGGGHTS code for generating DEM simulations.

## Appendix A. Analytical Projection of a Set of Spheres

Let the sphere, with centre $c_{p}={\left[c_{px},c_{py},c_{pz}\right]}^{T}$ and radius $r_{p}$, be defined by a scalar real valued analytical function, $s_{p}\left(x\right)$, in 3D real space, $x={\left[x,y,z\right]}^{T}$, such that

(A1) $$\begin{array}{cc} \; & s_{p}\left(x\right)=\left\{\begin{array}{c} \rho_{p}\;\mathrm{if}\;{\left(x-c_{p}\right)}^{T}\left(x-c_{p}\right)\leq r_{p}^{2}\\ 0\;\mathrm{otherwise} \end{array}\right.. \end{array}$$

We define the origin of the Cartesian coordinate system and define it by the symbol $O_{r}$. Next we introduce a point-like X-ray source at $O_{s}$ and let a projection plane go through the point $O_{d}$ with normal $\hat{d}=\left(O_{s}-O_{d}\right)/\left|\right|\left(O_{s}-O_{d}\right){\left|\right|}_{2}$. Considering a single point in the detector plane, $P_{d}$, we seek to compute the intersection of a ray parallel to $\hat{n}=\left(P_{d}-O_{s}\right)/\left|\right|P_{d}-O_{s}{\left|\right|}_{2}$ with the sphere $s_{p}$. Since spheres are convex objects this is easily done by considering the two cases of intersection or no intersection. If an intersection exist the intersection points are given as

(A2) $${\left(x-c_{p}\right)}^{T}\left(x-c_{p}\right)=r^{2},\;x=O_{s}+s\hat{n}.$$

Expansion yields

(A3) $$s^{2}+2s{\hat{n}}^{T}\left(O_{s}-c_{p}\right)+O_{s}^{T}O_{s}+c_{p}^{T}\left(c_{p}-2O_{s}\right)-r^{2}=0.$$

Introducing the scalar

(A4) $$S_{p}={\left({\hat{n}}^{T}\left(O_{s}-c_{p}\right)\right)}^{2}-O_{s}^{T}O_{s}+c_{p}^{T}\left(2O_{s}-c_{p}\right)+r^{2},$$

the intersections are given as

(A5) $$s={\hat{n}}^{T}\left(c_{p}-O_{s}\right)\pm \sqrt{S_{p}}.$$

From this we can see that the ray path length through the sphere is

(A6) $$L_{p}=2\sqrt{S_{p}}.$$

For the case when no intersection exists, the value under the root will be negative. Thus, we may compactly write the general attenuation contribution from a sphere as a real part,

(A7) Ap=2ρpReSp.

Letting the value of $s_{p}$ represent the sphere attenuation, we have that the analytical projection of a set of spheres at arbitrary detector location is

(A8) A=∑p=1p=P2ρpRe2Sp.

Introducing the rotation axis $\hat{z}$ and the angle θ as a positive sample rotation, we may compute the projection at any rotation by setting

(A9) $$\begin{array}{cc} \; & O_{s}=R_{z}\left[\begin{array}{c} -S\\ 0\\ 0 \end{array}\right],\;O_{d}=R_{z}\left[\begin{array}{c} D\\ 0\\ 0 \end{array}\right],\\ \; & \hat{n}=\frac{1}{\sqrt{{\left(D+S\right)}^{2}+x_{d}^{2}+y_{d}^{2}}}R_{z}\left[\begin{array}{c} D+S\\ x_{d}\\ y_{d} \end{array}\right]. \end{array}$$

Here *S* and *D* are the source to rotation axis and detector to rotation axis distances, respectively, and $x_{d},y_{d}$ are the *x* and *y* coordinates of the detector ray intersection point. In this setting the line connecting the X-ray source, $O_{s}$, with the detector origin, $O_{d}$, is parallel to $\hat{x}$, i.e., the optical axis of propagation is along $\hat{x}$.

On a computational side, we mention here that the rendering time of projections may be reduced by only evaluating the root expression in (A8) for detector pixels that are inside a projected bounding rectangle associated with the considered particle.

## Appendix B. Derivation of Partial Derivatives of Flow Model

To enable solving of

(A10) $${\mathrm{Argmin}}_{\alpha_{j}}\sum_{k}{\left(P_{k}\left[\mathrm{FV}\left[\bar{f},u\right]\right]-\frac{\partial A_{k}\left(t_{l}\right)}{\partial t}\right)}^{2}$$

by numerical iterative schemes we seek to derive expressions for the partial derivatives $\partial C/\partial \alpha_{j}$ where we define the function, C, as

(A11) $$C\left(\alpha_{j}\right)=\sum_{k}{\left(P_{k}\left[\mathrm{FV}\left[\bar{f},u\right]\right]-\frac{\partial A_{k}\left(t_{l}\right)}{\partial t}\right)}^{2}.$$

Considering that the projection operator, $P_{k}\left[\cdot \right]$, is linear, we find that

(A12) $$\frac{\partial C}{\partial \alpha_{j}}=2\sum_{k}P_{k}\left[\frac{\partial \mathrm{FV}[\bar{f},u]}{\partial \alpha_{j}}\right]\left(P_{k}\left[\mathrm{FV}\left[\bar{f},u\right]\right]-\frac{\partial A_{k}\left(t_{l}\right)}{\partial t}\right).$$

Equation (A12) indicates that $P_{k}\left[\cdot \right]$ needs to be computed three times for each included basis function in the velocity expansion. Considering that the projection operation represents a computationally expensive ray-tracing algorithm, we select to rewrite (A12) to a backwards format as

(A13) $$\frac{\partial C}{\partial \alpha_{j}}=2\sum_{k}\frac{\partial \mathrm{FV}[\bar{f},u]}{\partial \alpha_{j}}P_{k}^{*}\left[P_{k}\left[\mathrm{FV}\left[\bar{f},u\right]\right]-\frac{\partial A_{k}\left(t_{l}\right)}{\partial t}\right],$$

where $P_{k}^{*}\left[\cdot \right]$ is the adjoint of $P_{k}\left[\cdot \right]$, commonly refered to as the "back-projection" operator. The equality of the backwards (A13) and forwards (A12) formulation follows from the fact that the enclosing sum goes over all k, defining a scalar product. With (A13), ray-tracing needs only be executed two times during gradient computation.

To complete the expression given in (A13), we seek now $\partial F\left[\bar{f},u\right]/\partial \alpha_{j}$. Considering the used flow scheme represented by (13), we find that the derivative of the *D*-th component (corresponding to dimensions x,D=1, y,D=2 or z,D=3) of the *j*-th velocity basis vector is

(A14) $$\begin{array}{cc} \; & \frac{F[\bar{f},u]}{\partial \alpha_{Dj}}=\frac{\partial }{\partial \alpha_{Dj}}\left(-\frac{1}{\Delta x}\sum_{d=1}^{d=3}\left(F_{di+}-F_{di-}\right)\right)=\\ \; & \frac{1}{\Delta x}\left(\sum_{d=1}^{d=3}\left(\frac{\partial F_{di-}}{\partial \alpha_{Dj}}-\frac{\partial F_{di+}}{\partial \alpha_{Dj}}\right)\right). \end{array}$$

With (14), the derivatives of the numerical fluxes at the cell faces setting d=D become

(A15) $$\begin{array}{cc} \; & \frac{\partial F_{di-}}{\partial \alpha_{Dj}}=\frac{1}{2}\left(\frac{\partial u_{Di-}}{\partial \alpha_{Dj}}\right)\left(f_{Di-}^{R}+f_{Di-}^{L}\right)-\\ \; & \frac{1}{2}\left(\frac{\partial |u_{Di-}|}{\partial \alpha_{Dj}}\right)\left(f_{Di-}^{R}-f_{Di-}^{L}\right),\\ \; & \frac{\partial F_{di+}}{\partial \alpha_{Dj}}=\frac{1}{2}\left(\frac{\partial u_{Di+}}{\partial \alpha_{Dj}}\right)\left(f_{Di+}^{R}+f_{Di+}^{L}\right)-\\ \; & \frac{1}{2}\left(\frac{\partial |u_{Di+}|}{\partial \alpha_{Dj}}\right)\left(f_{Di+}^{R}-f_{Di+}^{L}\right). \end{array}$$

For d≠D the derivatives vanishes

(A16) $$\begin{array}{cc} \; & \frac{\partial F_{di-}}{\partial \alpha_{Dj}}=0,\;\mathrm{if}\;d\neq D\\ & \frac{\partial F_{di+}}{\partial \alpha_{Dj}}=0,\;\mathrm{if}\;d\neq D \end{array}$$

Using the velocity basis decomposition defined in (22), we find that

(A17) $$\begin{array}{cc} \; & \frac{\partial u_{Di-}}{\partial \alpha_{Dj}}=\sum_{l=1}^{l=M}\left(\frac{\partial \alpha_{Dl}}{\partial \alpha_{Dj}}\right)\varphi_{l}\left(x_{Di-}\right)=\varphi_{j}\left(x_{Di-}\right),\\ \; & \frac{\partial |u_{Di-}|}{\partial \alpha_{Dj}}=\sum_{l=1}^{l=M}\left(\frac{\partial |\alpha_{Dl}|}{\partial \alpha_{Dj}}\right)\varphi_{l}\left(x_{Di-}\right)=\mathrm{sign}\left(\alpha_{Dj}\right)\varphi_{j}\left(x_{Di-}\right)\\ \; & \frac{\partial u_{Di+}}{\partial \alpha_{Dj}}=\sum_{l=1}^{l=M}\left(\frac{\partial \alpha_{Dl}}{\partial \alpha_{Dj}}\right)\varphi_{l}\left(x_{Di+}\right)=\varphi_{j}\left(x_{Di+}\right),\\ \; & \frac{\partial |u_{Di+}|}{\partial \alpha_{Dj}}=\sum_{l=1}^{l=M}\left(\frac{\partial |\alpha_{Dl}|}{\partial \alpha_{Dj}}\right)\varphi_{l}\left(x_{Di+}\right)=\mathrm{sign}\left(\alpha_{Dj}\right)\varphi_{j}\left(x_{Di+}\right) \end{array}$$

where sign(·) is the signum function and $\alpha_{Dj}\neq 0$. Insertion of (A17) into (A15) now yields

(A18) $$\begin{array}{cc} \; & \frac{\partial F_{di-}}{\partial \alpha_{Dj}}=\frac{1}{2}\varphi_{j}\left(x_{Di-}\right)\left(f_{di-}^{R}+f_{di-}^{L}\right)-\\ \; & \frac{1}{2}\mathrm{sign}\left(\alpha_{Dj}\right)\varphi_{j}\left(x_{Di-}\right)\left(f_{di-}^{R}-f_{di-}^{L}\right)\\ \; & \frac{\partial F_{di+}}{\partial \alpha_{Dj}}=\frac{1}{2}\varphi_{j}\left(x_{Di+}\right)\left(f_{di+}^{R}+f_{di+}^{L}\right)-\\ \; & \frac{1}{2}\mathrm{sign}\left(\alpha_{Dj}\right)\varphi_{j}\left(x_{Di+}\right)\left(f_{di+}^{R}-f_{di+}^{L}\right) \end{array}$$

Insertion of (A18) and (A16) into (A14) gives

(A19) $$\begin{array}{cc} \; & \frac{F[\bar{f},u]}{\partial \alpha_{Dj}}=\frac{1}{2\Delta x}[\varphi_{j}\left(x_{Di-}\right)\left(f_{Di-}^{R}+f_{Di-}^{L}\right)-\\ & \mathrm{sign}\left(\alpha_{Dj}\right)\varphi_{j}\left(x_{Di-}\right)\left(f_{Di-}^{R}-f_{Di-}^{L}\right)-\\ \; & \varphi_{j}\left(x_{Di+}\right)\left(f_{Di+}^{R}+f_{Di+}^{L}\right)+\\ & \mathrm{sign}\left(\alpha_{Dj}\right)\varphi_{j}\left(x_{Di+}\right)\left(f_{Di+}^{R}-f_{Di+}^{L}\right)]. \end{array}$$

With the additional definition that sign(0)=1, we find that Equation (A19), together with (A13), completely defines the sought derivatives.
