# Supplementary material for: A Continuity Flow Based Tomographic Reconstruction Algorithm for 4D Multi-Beam High Temporal—Low Angular Sampling
Source: J Imaging. 2021 Nov 20;7(11):246. doi: 10.3390/jimaging7110246 (PMC8624918; doi:10.3390/jimaging7110246)
Supplement: Supplementary file 1 [file jimaging-07-00246-s001.zip › jimaging-1437892-supplementary.pdf]

Supplementary Materials: A Continuity Flow Based Tomographic Reconstruction Algorithm for 4D Multi-Beam High Temporal–Low Angular Sampling

Axel Henningsson *,†,‡, Stephen A. Hall ‡

1. Comparison to existing iterative methods

To provide additional evaluation of the quality of the reconstructions produced by the proposed scheme (algorithm 1) we provide in this supplementary material a comparison to results obtainable by existing iterative reconstructions schemes. The reconstructions obtained by using algorithm 1 (referred to as "Flow" reconstruction in the following) has been compared to the results of ART, SIRT and TV as implemented in the open source library tomopy [1]. As can be seen in Figures S1-S3 comparisons was performed for all three phantom scenarios presented in the original paper. For an equidistant range of selected time-points the central volume slice was reconstructed providing attenuation fields in the $x - y$-plane. The reconstructions where compared to ground truth reference reconstructions found by using a full range of angular projections as described in more detail in the original papaer. The Root Mean Squared Error (RMSE) and Mean Absoloute Errors (MAE) where computed for the reconstructed slices with respect to the ground truth and plotted as functions of time for the various reconstruction methods. These error evolution plots are displayed in the bottom rows of Figures S1-S3. The top two rows in the corresponding figures illustrate reconstructions found by the different methods for a fixed time-step.

To provide a fair comparison between the methods each of the three iterative methods have been provided with the reference reconstruction existing at $t = 0$ which was used as an an initial guess. Furthermore, since the TV reconstruction scheme has a user defined parameter defining how much total variation is allowed in the final reconstruction we must define a procedure to select this regularisation parameter. To ensure that we do not unjustly benefit our own Flow reconstruction method we have selected to optimise the TV parameter by minimising the RMSE with respect to the current reconstruction that would be obtained from a full scan. Although this information is not available in the targeted multi-beam setting the procedure serves to ensure that the TV reconstruction is performing maximally good reconstructions with respect to the RMSE.

All iterative reconstructions where performed with a maximum allowed number of iterations equal to 100. The TV smoothing parameter was optimised by grid -searching in the range $[0.001, 50]$ using a subsequently refined step-size resulting in an optimal value found within ± 0.1.

The results provided in the bottom row error plots of Figure S1-S3 show that the Flow reconstruction method produces reconstructions of similar quality of those obtained with an optimised TV with respect to MAE and RMSE. As expected TV reconstruction consistently produced better reconstructions compared to ART and SIRT throughout time and between phantom scenarios. One of the main benefits of using the Flow reconstruction scheme over TV lies in the disposal of the regularisation parameter. In the Flow reconstruction the regularisation is instead inherently applied through the inclusion of the continuity equations.

Turning to the 2D slice reconstructions of Figure S1-S3 one could argue that the Flow reconstructions are producing the best reconstructions from a qualitatively point of view. Especially for Figure S2 and S3 the Flow reconstruction has managed to preserve some of the granular structure surrounding the central impactor. In these cases both ART and SIRT seem to be content with a slight modification of their initial guess leaving little trace of the impactor in the final reconstruction. On the other hand TV manages to both capture the

impactor and the contours of the sample, however, all internal grain structure is lost. When making such qualitatively comparisons it should be remembered that the TV regularisation parameter was optimised with respect to RMSE and not by visual inspection. It is thus possible that some additional structure of the grains could also be revealed by the TV scheme at the cost of increasing the RMSE.

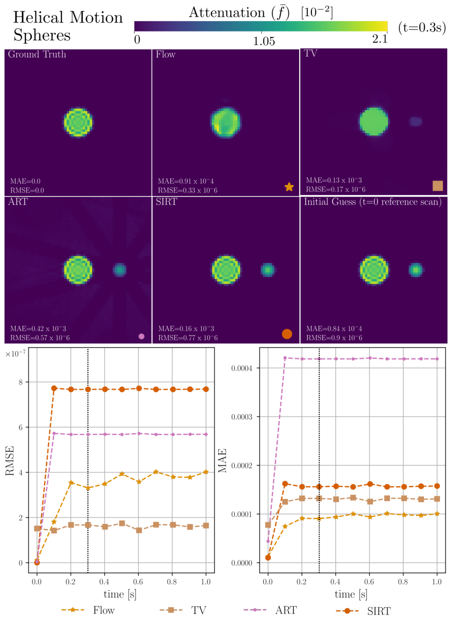

Figure S1. The top two rows depict reconstructions of a helical motion three body system by ART, SIRT, TV and the proposed Flow scheme. The central $x - y$ slice at $t = 0.3$s has been selected for display. The ground truth at $t = 0.3$s is displayed in the top left corner while the initial guess provided to ART, SIRT and TV is displayed in the bottom left corner. The bottom row plots show the temporal evolution of the RMSE and MAE as compared to ground truth for the different reconstructions. The vertical dashed line in the RMSE and MAE plots marks the time-point at which the top reconstructions originate form.

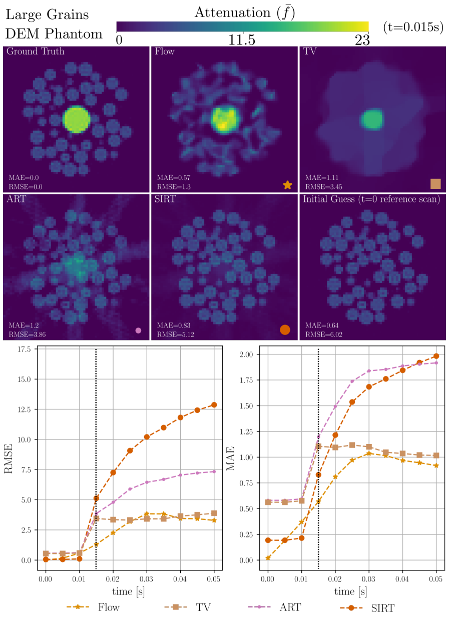

Figure S2. The top two rows depict reconstructions of a granular system (featuring small grains and a gravity driven impactor) by ART, SIRT, TV and the proposed Flow scheme. The central $x-y$ slice at $t = 0.015$s has been selected for display. The ground truth at $t = 0.015$s is displayed in the top left corner while the initial guess provided to ART, SIRT and TV is displayed in the bottom left corner. The bottom row plots show the temporal evolution of the RMSE and MAE as compared to ground truth for the different reconstructions. The vertical dashed line in the RMSE and MAE plots marks the time-point at which the top reconstructions originate form.

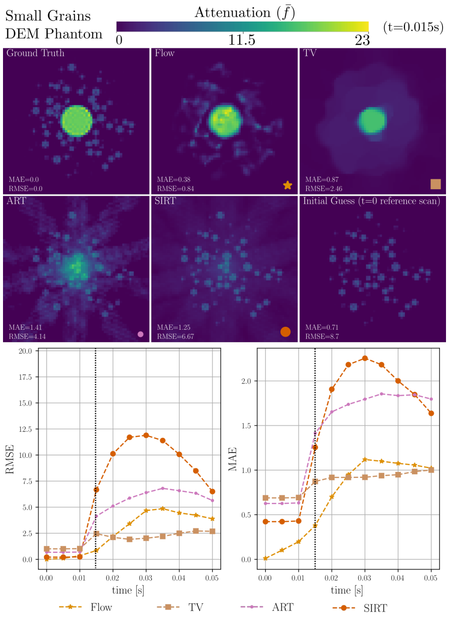

Figure S3. The top two rows depict reconstructions of a granular system (featuring small grains and a gravity driven impactor) by ART, SIRT, TV and the proposed Flow scheme. The central $x-y$ slice at $t = 0.015$s has been selected for display. The ground truth at $t = 0.015$s is displayed in the top left corner while the initial guess provided to ART, SIRT and TV is displayed in the bottom left corner. The bottom row plots show the temporal evolution of the RMSE and MAE as compared to ground truth for the different reconstructions. The vertical dashed line in the RMSE and MAE plots marks the time-point at which the top reconstructions originate form.

2. LIGGGHTS script for simulating impact upon granular bed

Here follows the code used to generate dynamic granular system phantoms with LIGGGHTS [2].

```
# The following code uses the LIGGGHTS software to simulate gravity driven impact upon a granular bed.

##################################################################################################
# Large grains distribution

### Initialization
# Preliminaries
units si
atom_style sphere
atom_modify map array
boundary f f f
newton off
communicate single vel yes

# Declare domain
region domain block -0.138 0.138 -0.138 0.138 -0.06 0.43 units box
create_box 2 domain

# Neighbor listing
neighbor 0.032 bin
neigh_modify every 1 check no
neigh_modify delay 0

### Setup
# Material and interaction properties required
fix m1 all property/global youngsModulus peratomtype 2.5e7 2.5e7
fix m2 all property/global poissonsRatio peratomtype 0.25 0.25
fix m3 all property/global coefficientRestitution peratomtypepair 2 0.5 0.5 0.5 0.5
fix m4 all property/global coefficientFriction peratomtypepair 2 0.2 0.175 0.175 0.5

# Particle distributions and insertion

# spheres
fix pts1 all particletemplate/sphere 15485863 atom_type 1 density constant 2650 radius constant 0.00325
fix pts2 all particletemplate/sphere 15485867 atom_type 1 density constant 2650 radius constant 0.00350
fix pts3 all particletemplate/sphere 32452843 atom_type 1 density constant 2650 radius constant 0.00375
fix pts4 all particletemplate/sphere 49979693 atom_type 1 density constant 2650 radius constant 0.00425
fix pts5 all particletemplate/sphere 49979687 atom_type 1 density constant 2650 radius constant 0.00450
fix pts6 all particletemplate/sphere 86028121 atom_type 1 density constant 2650 radius constant 0.00475
fix pts7 all particletemplate/sphere 86028157 atom_type 1 density constant 2650 radius constant 0.00500

# make a template
fix pdd all particledistribution/discrete/numberbased 32452867 7 &
pts1 0.1 pts2 0.1 pts3 0.15 pts4 0.3 pts5 0.15 pts6 0.1 pts7 0.1

# define the seedin region of the container
region seeding_region cylinder z 0 0 0.03 0.08 0.10 units box

# insert particles into the container
fix ins all insert/rate/region seed 67867979 distributiontemplate pdd &
nparticles 300 particlerate 20000 insert_every 50 &
overlapcheck yes vel constant 0. 0. -1.0 region seeding_region ntry_mc 10000

# Rigid cylinder + bottom plane
fix cylinderwall all wall/gran model hertz tangential history primitive type 1 zcylinder 0.04 0.0 0.0
fix stopper all wall/gran model hertz tangential history primitive type 2 zplane 0.0

# Define the physics
pair_style gran model hertz tangential history
pair_coeff * *

### Detailed settings
# Integrator
fix integrate all nve/sphere
fix integr all multisphere

# Gravity
fix grav all gravity 9.81 vector 0.0 0.0 -1.0

# Time step
timestep 0.00001

# Thermodynamic output settings
thermo_style custom step atoms ke cpu
thermo 1000
thermo_modify norm no lost ignore

# Check time step and initialize dump file
fix ctg all check/timestep/gran 1 0.01 0.01
run 1
unfix ctg

### Execution and further settings
# Fill the hopper
run 50000 upto

# Insert an impact bullet on the grain ensemble
fix impactpts all particletemplate/sphere 123457 atom_type 1 density constant 10600 radius constant 0.015
fix impactpdd all particledistribution/discrete 17903 1 impactpts 1.0
region impactregion cylinder z 0 0 0.001 0.04 0.08 units box

fix impactins all insert/pack seed 56543 distributiontemplate impactpdd vel constant 0. 0. -1.0 &
                insert_every once overlapcheck yes region impactregion ntry_mc 10000 particles_in_region 1

dump dmp all custom/vtk 10 post/impact_large_grains_*.vtk id type type x y z ix iy iz vx vy vz fx fy fz omegax omegay omegaz radius mass

run 5000

# end large grains distribution
##################################################################################################
```

```
##################################################################################################
# Small grains distribution

### Initialization
# Preliminaries
units si
atom_style sphere
atom_modify map array
boundary f f f
newton off
communicate single vel yes

# Declare domain
region domain block −0.138 0.138 −0.138 0.138 −0.06 0.43 units box
create_box 2 domain

# Neighbor listing
neighbor 0.020 bin
neigh_modify every 1 check no
neigh_modify delay 0

### Setup
# Material and interaction properties required
fix m1 all property/global youngsModulus peratomtype 2.5e7 2.5e7
fix m2 all property/global poissonsRatio peratomtype 0.25 0.25
fix m3 all property/global coefficientRestitution peratomtypepair 2 0.5 0.5 0.5 0.5
fix m4 all property/global coefficientFriction peratomtypepair 2 0.2 0.175 0.175 0.5

# Particle distributions and insertion

# spheres
fix pts1 all particletemplate/sphere 15485863 atom_type 1 density constant 2650 radius constant 0.00150
fix pts2 all particletemplate/sphere 15485867 atom_type 1 density constant 2650 radius constant 0.00175
fix pts3 all particletemplate/sphere 32452843 atom_type 1 density constant 2650 radius constant 0.00200
fix pts4 all particletemplate/sphere 49979693 atom_type 1 density constant 2650 radius constant 0.00225
fix pts5 all particletemplate/sphere 49979687 atom_type 1 density constant 2650 radius constant 0.00250
fix pts6 all particletemplate/sphere 86028121 atom_type 1 density constant 2650 radius constant 0.00275
fix pts7 all particletemplate/sphere 86028157 atom_type 1 density constant 2650 radius constant 0.00300

# make a template
fix pdd all particledistribution/discrete/numberbased  32452867 7 &
pts1 0.1 pts2 0.1 pts3 0.15 pts4 0.3 pts5 0.15 pts6 0.1 pts7 0.1

# define the seedin region of the container
region seeding_region cylinder z 0 0 0.03 0.08 0.10 units box

# insert particles into the container
fix ins all insert/rate/region seed 67867979 distributiontemplate pdd &
nparticles 2200 particlerate 20000 insert_every 50 &
overlapcheck yes vel constant 0. 0. −1.0 region seeding_region ntry_mc 10000

# Rigid cylinder + bottom plane
fix cylinderwall all wall/gran model hertz tangential history primitive type 1 zcylinder 0.04 0.0 0.0
fix stopper all wall/gran model hertz tangential history primitive type 2 zplane 0.0

# Define the physics
pair_style gran model hertz tangential history
pair_coeff * *

### Detailed settings
# Integrator
fix integrate all nve/sphere
fix integr all multisphere

# Gravity
fix grav all gravity 9.81 vector 0.0 0.0 −1.0

# Time step
timestep 0.00001

# Thermodynamic output settings
thermo_style custom step atoms ke cpu
thermo 1000
thermo_modify norm no lost ignore

# Check time step and initialize dump file
fix ctg all check/timestep/gran 1 0.01 0.01
run 1
unfix ctg

### Execution and further settings
# Fill the hopper
run 50000 upto

# Insert an impact bullet on the grain ensemble
fix impactpts all particletemplate/sphere 123457 atom_type 1 density constant 10600 radius constant 0.015
fix impactpdd all particledistribution/discrete 17903 1 impactpts 1.0
region impactregion cylinder z 0 0 0.001 0.04 0.08 units box

fix impactins all insert/pack seed 56543 distributiontemplate impactpdd vel constant 0. 0. −1.0 &
                insert_every once overlapcheck yes region impactregion ntry_mc 10000 particles_in_region 1

dump dmp all custom/vtk 10 post/impact_small_grains_*.vtk id type type x y z ix iy iz vx vy vz fx fy fz omegax omegay omegaz radius mass

run 5000

# end small grains distribution
##################################################################################################
```

References

1. Gürsoy, D.; De Carlo, F.; Xiao, X.; Jacobsen, C. Tomopy: A framework for the analysis of synchrotron tomographic data. *J. Synchrotron Radiat.* **2014**, *21*, 1188–1193.
2. Kloss, C.; Goniva, C.; Hager, A.; Amberger, S.; Pirker, S. Models, algorithms and validation for opensource DEM and CFD–DEM. *Prog. Comput. Fluid Dyn. Int. J.* **2012**, *12*, 140–152. doi:10.1504/PCFD.2012.047457.
